# Multiple Loci Associated with Renal Function in African Americans

**DOI:** 10.1371/journal.pone.0045112

**Published:** 2012-09-13

**Authors:** Daniel Shriner, Alan Herbert, Ayo P. Doumatey, Jie Zhou, Hanxia Huang, Michael R. Erdos, Guanjie Chen, Norman P. Gerry, Michael F. Christman, Adebowale Adeyemo, Charles N. Rotimi

**Affiliations:** 1 Center for Research on Genomics and Global Health, National Human Genome Research Institute, Bethesda, Maryland, United States of America; 2 Department of Genetics and Genomics, Boston University School of Medicine, Boston, Massachusetts, United States of America; 3 Genome Technology Branch, National Human Genome Research Institute, Bethesda, Maryland, United States of America; 4 The Coriell Institute for Medical Research, Camden, New Jersey, United States of America; The University of Texas Health Science Center (UTHSCSA ), United States of America

## Abstract

The incidence of chronic kidney disease varies by ethnic group in the USA, with African Americans displaying a two-fold higher rate than European Americans. One of the two defining variables underlying staging of chronic kidney disease is the glomerular filtration rate. Meta-analysis in individuals of European ancestry has identified 23 genetic loci associated with the estimated glomerular filtration rate (eGFR). We conducted a follow-up study of these 23 genetic loci using a population-based sample of 1,018 unrelated admixed African Americans. We included in our follow-up study two variants in *APOL1* associated with end-stage kidney disease discovered by admixture mapping in admixed African Americans. To address confounding due to admixture, we estimated local ancestry at each marker and global ancestry. We performed regression analysis stratified by local ancestry and combined the resulting regression estimates across ancestry strata using an inverse variance-weighted fixed effects model. We found that 11 of the 24 loci were significantly associated with eGFR in our sample. The effect size estimates were not significantly different between the subgroups of individuals with two copies of African ancestry *vs*. two copies of European ancestry for any of the 11 loci. In contrast, allele frequencies were significantly different at 10 of the 11 loci. Collectively, the 11 loci, including four secondary signals revealed by conditional analyses, explained 14.2% of the phenotypic variance in eGFR, in contrast to the 1.4% explained by the 24 loci in individuals of European ancestry. Our findings provide insight into the genetic basis of variation in renal function among admixed African Americans.

## Introduction

Chronic kidney disease (CKD) is a common health condition that increases the risk of kidney failure and cardiovascular disease. According to two National Health and Nutrition Examination Surveys, the prevalence of CKD in the USA increased from 10.0% in 1988–1994 to 13.1% in 1999–2004 [1]. Particularly alarming is the projection that the public health burden of CKD will worsen due to the increasing prevalence in the general population of major risk factors including hypertension, obesity, and type 2 diabetes [1], [2]. The risk of developing CKD varies by ethnicity, with African Americans having about two-fold higher risk than European Americans after controlling for clinical, lifestyle, and socio-demographic factors [3]. Stages of CKD are defined by a combination of renal damage, often quantified by proteinuria, and renal function, often quantified by the estimated glomerular filtration rate (eGFR) [2].

There is strong evidence for genetic loci influencing eGFR. In the Framingham Heart Study, the heritability estimate for eGFR was 0.46 [4]. In families of European ancestry ascertained for type 2 diabetes, the estimated heritability for eGFR was 0.75 [5]. In a genome-wide association study of European-ancestry individuals, genetic variants in the genes *UMOD*, *SHROOM3*, and *GATM*-*SPATA5L1* were associated with eGFR [6]. Associations at these three loci explained 0.7% of the phenotypic variance of eGFR, indicating the presence of additional genetic risk factors [6]. Meta-analysis has revealed a total of 23 loci influencing eGFR, collectively explaining 1.4% of the phenotypic variance of eGFR among individuals of European ancestry [7]. Using admixture mapping, *APOL1* has been identified as a risk gene for non-diabetic end-stage renal disease in African Americans [8], [9], [10]; whether this gene is also associated with eGFR is unknown.

We performed a follow-up study of these 24 loci for association with eGFR in admixed African Americans. Our main objectives were to assess transferability of reported genetic associations originally detected in individuals of European ancestry to our sample of admixed African Americans and to test if *APOL1* influences eGFR. We used stratified regression to account for confounding of association by ancestry at the level of the individual as well as the marker [11]. For those associations that transferred to our sample, we used differences in linkage disequilibrium patterns to fine-map the loci and we used conditional analysis to detect secondary associations [12].

## Results

We assessed the transferability of association with eGFR for 23 index single nucleotide polymorphisms (SNPs) from European ancestry to admixed African Americans. Our sample of 1,018 unrelated individuals (Table 1) provided at least 80% power to detect effects explaining ≥0.77% of the phenotypic variance at the 5% significance level [13]. For each of the 23 loci, we identified a set of proxies for the index SNP based on the HapMap Phase II+III CEU reference data. Our strategy revealed that the most strongly associated SNP in our sample differed from the index SNP for each of the 23 loci (Table 2). We detected significant association for 10 of the 23 loci (Table 2 and Supplementary Figure S1). Collectively, these 10 loci explained 8.8% of the phenotypic variance of eGFR in our sample (Supplementary Table S1).

**Table 1:** Characteristics of the study sample.

Variable	Value
Sample size	1,018
Age (years)	48 (41, 56)^a^
Male:Female	419:599
Percent African ancestry	82.0 (74.7, 87.5)
Serum creatinine (mg/dL)	0.9 (0.7, 1.0)
Glomerular filtration rate (mL/min/1.73 m^2^)	102.1 (86.7, 119.2)
Type 2 diabetes (prevalence)	15.6%
Hypertension (prevalence)	50.0%

a Ranges are presented as median (first quartile, third quartile).

**Table 2:** Association with eGFR stratified by ancestry.

Index SNP	Chr	Position^a^	Gene	No. SNPs^b^	DoF^c^	BestSNP	Position	CodedAllele	β_meta_ d	SE_meta_ d	Adjusted *P*	*F_ST_*	iHS_CEU_	iHS_YRI_
rs1933182	1	109801361	*SYPL2*	31	3.62	rs12136063	109815693	G	0.426	0.166	0.037	0.246	−0.425	0.746
rs267734	1	149218101	*ANXA9*	40	6.63	rs3754210	149220885	G	−0.296	0.152	0.336	0.084	−0.163	−1.136
rs1260326	2	27584444	*GCKR*	41	6.28	rs13392197	27657618	C	−0.688	0.386	0.472	0.015	NA	−1.079
rs13538	2	73721836	*NAT8*	56	4.79	rs2567603	73839633	G	0.846	0.383	0.130	NA	1.867	NA
rs7422339	2	211248752	*CPS1*	35	4.65	rs2287413	211325773	C	0.573	0.351	0.476	0.055	−1.404	NA
rs347685	3	143289827	*TFDP2*	33	5.04	rs11569291	143152375	A	−1.670	0.575	0.019	NA	0.322	NA
rs17319721	4	77587871	*SHROOM3*	74	8.98	rs10025494	77408404	A	0.372	0.159	0.173	0	−0.480	0.801
rs11959928	5	39432889	*DAB2*	26	4.70	rs700242	39420645	A	−0.255	0.161	0.531	0.035	0.029	−0.128
rs6420094	5	176750242	*SLC34A1*	24	4.99	rs10037055	176623885	G	0.381	0.147	0.049	0.237	−0.552	0.171
rs881858	6	43914587	*VEGFA*	9	4.62	rs744103	43913340	T	0.213	0.163	0.884	0.281	0.937	1.984
rs2279463	6	160588379	*SLC22A2*	42	8.33	rs2774225	160621171	G	0.559	0.147	1.16×10^−3^	0.004	−1.747	0.648
rs6465825	7	77254375	*PHTF2*	56	6.52	rs12705112	77380997	C	−1.473	0.511	0.026	NA	−0.786	NA
rs4744712	9	70624527	*PIP5K1B/* *FAM122A*	103	8.89	rs17482181	70577820	C	0.705	0.178	6.32×10^−4^	0.031	−1.315	−0.109
rs10794720	10	1146165	*WDR37*	38	6.73	rs6560711	1158563	A	−0.410	0.180	0.156	0.009	0.711	1.591
rs4014195	11	65263398	*OVOL1*	37	6.22	rs489574	65299315	G	0.469	0.170	0.035	0.006	−0.358	−0.025
rs10774021	12	219559	*SLC6A13*	35	6.18	rs486098	258918	C	0.349	0.170	0.245	0.020	−0.507	−1.843
rs626277	13	71245697	*DACH1*	21	4.38	rs9564840	71235100	A	−0.759	0.412	0.287	NA	0.648	NA
rs2467853	15	43486085	*SLC30A4*	72	4.85	rs6493153	43561217	A	−0.516	0.162	0.007	NA	NA	−1.637
rs491567	15	51733885	*WDR72*	98	9.46	rs4332691	51677654	C	0.929	0.289	0.012	0.198	−1.380	NA
rs1394125	15	73946038	*UBE2Q2*	23	4.16	rs335711	73971795	C	−0.232	0.160	0.614	0.040	−0.840	−2.534
rs12917707	16	20275191	*UMOD*	14	4.43	rs4522429	20293616	C	0.260	0.161	0.472	0.113	0.790	0.441
rs9895661	17	56811371	*BCAS3/TBX2*	15	4.17	rs11079428	56821483	T	0.535	0.166	0.005	0.467	−1.631	0.289
rs12460876	19	38048731	*SLC7A9*	63	7.99	rs17272267	38063033	G	−0.713	0.280	0.086	0.087	−2.373	NA

a Positions are based on NCBI build 36.

b Shown are the nominal numbers of SNPs in the set for each locus.

c “DoF” indicates the effective degrees of freedom for each locus, which is the correction factor used to adjust *p*-values.

d β_meta_ and SE_meta_ refer to the estimates from the meta-analysis combined across the three strata of local ancestry.

Given that the 8.8% of the phenotypic variance explained in our sample was considerably larger than the 1.4% explained among individuals of European ancestry [7], we performed a reality check by estimating the percent variance explained when comparing a full model consisting of the 10 SNPs, age, sex, and global ancestry with a reduced model consisting of age, sex, and global ancestry. Uncorrected for local ancestry, genotypic effects of our 10 most strongly associated SNPs collectively explained 5.7% of the phenotypic variance of eGFR. By comparison, genotypic effects of the 10 index SNPs explained only 1.1% of the phenotypic variance of eGFR. Thus, even without accounting for local ancestry, our fine-mapping strategy enabled explanation of four-fold more phenotypic variance in our African American sample with less than half of the loci discovered in the meta-analysis of individuals of European ancestry. The 10 local ancestries collectively explained an additional 2.0% of the phenotypic variance. Principal component analysis revealed only one significant principal component, reflecting global ancestry and indicating an absence of residual population structure (Supplementary Figure S2). There was no compelling evidence for recent positive natural selection at any of the loci in either of the ancestral samples (Table 2). Overall weaker levels of linkage disequilibrium in African Americans compared to Europeans improved resolution of associated regions from an average of 199.7 kb to 15.0 kb (Supplementary Figure S1).

We also tested two markers in *APOL1* previously associated with end-stage renal disease [10]. The SNP rs73885319 was not associated with eGFR under either the additive (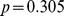) or recessive (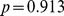) model. The 6 bp deletion rs71785313 was associated with eGFR under the additive (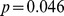) but not recessive (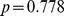) model. Rs71785313 showed an excess of heterozygotes (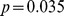). The association at rs71785313 explained an additional 0.3% of the phenotypic variance (Supplementary Table S1). Both markers are monomorphic in individuals of European ancestry and hence explain no phenotypic variance in such individuals.

To further explore the effects of local ancestry, we tested the allele frequencies in the subgroups of 0 *vs*. 2 chromosomes of African ancestry. The allele frequencies were statistically different at 10 of the 11 loci (Supplementary Table S2). This amount of differentiation for 11 loci was not excessive, given the amount of differentiation genome-wide between the CEU and YRI reference samples (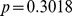). The effect size estimates were not statistically different between the two subgroups at any of the 11 loci (Supplementary Table S2). Similarly, none of the 11 loci showed an admixture effect, *i.e.*, an effect of excess ancestry (Supplementary Table S2).

We performed secondary analyses for the 10 loci discovered in individuals of European ancestry using the same stratified regression approach. We tested each SNP within a locus, conditioned on the most strongly associated SNP within that locus. We detected four secondary associations at three loci, *PSMA5*, *TFDP2*, and *WDR72*, collectively explaining an additional 3.1% of the phenotypic variance of eGFR (Supplementary Table S3). Taken together, genotype effects at the 11 associated loci explained 8.8%+3.1%+0.3% = 12.2% of the phenotype variance, with local ancestry effects explaining an additional 2.0% of the phenotypic variance, compared to 1.4% explained by all 24 loci in individuals of European ancestry.

The stratified regression approach can also be used as is at the genome-wide scale. None of the 797,831 SNPs was genome-wide significant for association with eGFR using either the unstratified or stratified approach (Supplementary Figure S3).

## Discussion

Association analysis can be confounded by variation in ancestry. Similar confounding can result from population structure among discrete populations, *i.e.*, multiple ancestrally homogeneous populations, as well as population structure due to admixture [14]. We recently developed a stratified regression approach for association analysis that controls by individual and, critically for admixed samples, by marker, for confounding due to variation in ancestry [11], [15]. We applied this approach in a follow-up study for eGFR in admixed African Americans given associations originally discovered in individuals of European ancestry. Despite substantial heterogeneity in allele frequencies, we found significant associations for 10 of 23 loci (*SYPL2*, *TFDP2*, *SLC34A1*, *SLC22A2*, *PHTF2*, *PIP5K1B*/*FAM122A*, *OVOL1*, *SLC30A4*, *WDR72*, and *BCAS3*/*TBX2*). Furthermore, we fine-mapped the associations at the 10 loci by leveraging the generally weaker levels of linkage disequilibrium in individuals of African ancestry compared to individuals of European ancestry, improving resolution by over an order of magnitude on average. We also found association with eGFR for a deletion in *APOL1* originally associated with end-stage renal disease.

Allele frequencies were significantly differentiated between African and European ancestries for 10 of the 11 loci. Given the amount of differentiation between the CEU and YRI samples at the genome-wide level, 10 of 11 loci showing significant allelic differentiation is not excessive. We found no evidence supporting the action of recent positive natural selection at the 10 loci discovered in individuals of European ancestry, suggesting that differentiation at the 10 loci reflects divergence due to random genetic drift following the ancestral split between Africans and Europeans. In contrast, evidence for balancing selection exists at *APOL1* [10].

We found that stratifying genotypes by local ancestry resulted in an additional 3.0% of phenotypic variance explained over genotypes uncorrected for local ancestry, indicating that local ancestry masked genotypic effects. Thus, accounting for local ancestry can increase the phenotypic variance explained by two distinct mechanisms: the direct effect of local ancestry, which is the basis of admixture mapping, and the indirect effect of masking genotypic effects, which is revealed by removing confounding. Previously, associations at 12/23 loci were reported to transfer to African Americans, but those results did not account for local ancestry and therefore are potentially subject to confounding [16].

There are three main limitations of our study. One limitation is that the HUFS sample of 1,018 unrelated individuals has less than 80% power in a follow-up study to detect effects explaining 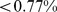 of the phenotypic variance and is underpowered in a discovery study for a polygenic trait. Another limitation is a lack of data addressing how the implicated genes influence eGFR. Possibilities include genes that affect creatinine metabolism (*SLC22A2*, *PHTF2*, *WDR72*, and *BCAS3*/*TBX2*) and genes that affect renal function (*SYPL2*, *TFDP2*, *SLC34A1*, *PIP5K1B*/*FAM122A*, *OVOL1*, and *SLC30A4*) [7]. The third limitation is that we did not include imputation, which is used to increase coverage of the genome by predicting genotypes for untyped markers. Imputation is based on the conditional distribution of haplotype frequencies given external reference data and thus requires specification of local ancestry. For admixed individuals such as African Americans, it is necessary to account for variation in ancestry both by individual and by marker and methods to do so are under development.

In summary, we identified 11 genetic loci influencing eGFR in a population-based sample of African Americans. We used stratified regression to test for association conditional on local ancestry, thereby controlling for confounding due to variation in ancestry at the individual level as well as at the marker level. We showed that the effect size estimates were population-consistent across the subgroups of individuals with two chromosomes of African ancestry *vs*. two chromosomes of European ancestry but that allele frequencies differed substantially. Our fine-mapping approach identified SNPs more strongly associated with eGFR than the index SNPs originally discovered in individuals of European ancestry. In addition, we demonstrated that local ancestry can mask genotypic effects. Consequently, our approach explained an increased proportion of phenotypic variance. Taken together, 11 loci explained 14.2% of the variance in eGFR. The approach implemented in this study can explain some of the missing heritability by simultaneously identifying more strongly associated SNPs, controlling for confounding due to ancestry, and identifying secondary associations, thereby enhancing disease and trait locus mapping in admixed as well as ancestrally homogenous populations.

## Materials and Methods

Ethics approval for the Howard University Family Study (HUFS) was obtained from the Howard University Institutional Review Board and written informed consent was obtained from each participant. All clinical investigation was conducted according to the principles expressed in the Declaration of Helsinki.

The HUFS is a population-based genetic epidemiology study of African Americans in the Washington, D.C. metropolitan area. We analyzed 1,018 unrelated individuals [17]. Using the Affymetrix Genome-Wide Human SNP Array 6.0 and established quality control filters, we obtained genome-wide genotypes for 808,465 autosomal single nucleotide polymorphisms (SNPs) [17]. In order to address possible confounding due to variation in ancestry, we first estimated the local ancestry (0, 1, or 2 chromosomes of African ancestry) for 797,831 unique autosomal SNPs using LAMPANC version 2.3 [18] and HapMap Phase II+III CEU and YRI reference allele frequencies (http://hapmap.ncbi.nlm.nih.gov/downloads/frequencies/2010-08_phaseIIIII/). Genome-wide, the average proportion of African ancestry was 0.799 (Supplementary Figure S4). Individual admixture proportions or global ancestry calculated from these estimates of local ancestry correlated very highly (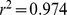) with estimates from STRUCTURE [19] using a panel of 2,076 ancestrally informative markers [20]. Our approach improves upon previous methodology [21] by assessing local ancestry for every genotyped marker rather than relying on local ancestry estimates at a small number of ancestrally informative markers and extrapolation of local ancestry for other markers.

Recognizing that the originally discovered index SNP might not be the most strongly associated SNP in follow-up samples, we followed a previously described fine-mapping strategy [12]. First, a region of linkage disequilibrium (LD) surrounding the index SNP was defined with bounds determined by the SNPs most distant to the index SNP with *r* ^2^≥0.3 using the HapMap CEU LD data (http://hapmap.ncbi.nlm.nih.gov/downloads/ld_data/latest/). Second, we estimated the covariance matrix for this set of SNPs using our sample genotypes. Third, this covariance matrix was spectrally decomposed and the effective degrees of freedom, *N_eff_*, was estimated using the relationship 
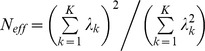
, in which 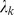 is the *k* ^th^ eigenvalue of the *K*×*K* covariance matrix for the *K* SNPs in the set [22]. For the locus, the significance threshold was set at 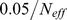. We did not correct for multiple loci because testing multiple loci in a follow-up study does not constitute a family of tests [23].

Serum creatinine levels were estimated on fasting samples using the modified Jaffé method. eGFR was calculated using the simplified Modification of Diet in Renal Disease Study equation [2], [24]: (eGFR (ml/min/1.73 m^2^) = 186×(serum creatinine)^−1^.^154^×age^−0^.^203^ (×0.742 if female) (×1.210 if black). Due to non-normality, the distribution of eGFR was transformed using a Box-Cox transformation (Supplementary Figure S5). We winsorized the distribution at ±3 standard deviations to reduce residual positive kurtosis. We then performed linear regression for eGFR on genotype assuming an additive genetic model, stratified by local ancestry and adjusted for age, sex, and global ancestry [11], following the model used in the original publications (in which neither hypertension nor type 2 diabetes were significant covariates) [6], [7]. We reported effect size estimates oriented with respect to 0, 1, or 2 copies of the allele listed as “coded.” Covariate adjustment for local ancestry assumes that ancestry effects are additive whereas our stratification approach is model-free in this regard. Covariate adjustment for local ancestry also assumes that local ancestry and genotype are independent, whereas local ancestry and genotype are known to covary [25], [26], [27]. Our stratification approach is based on the fact that genotype cannot be confounded by local ancestry within a homogeneous stratum of local ancestry. Finally, we combined the three sets of regression coefficients and standard errors using inverse variance-weighted fixed effects meta-analysis [28]. The fixed effect model assumes that inferential interest is limited to the observed local ancestry strata whereas the random effects model assumes that the observed local ancestry strata are part of a larger set of possible strata. Given that our study design limits local ancestry to three possible strata (*i.e.*, 0, 1, or 2 chromosomes of African ancestry), the fixed effects model is more appropriate. We performed conditional analysis for associated loci by following the same stratified regression approach, with the addition of an additive covariate for the genotype of the most strongly associated SNP within the locus. Regression analysis was performed using R [29].

To test whether 10/11 SNPs showing significant allelic differentiation was excessive, we randomly sampled without replacement11 SNPs from the 797,831 SNPs included in our study. For each SNP, we performed a test of proportions using the HapMap CEU and YRI allele counts. We estimated a *p*-value by recording how many times out of 10,000 independent replicates ≥10/11 SNPs showed allelic differentiation.

Principal components analysis of the genome-wide data was performed to estimate how much genetic variance was explained by admixture [30]. Briefly, autosomal SNPs were pruned for linkage disequilibrium at 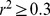 and then randomly thinned to 10,000 SNPs. These data were merged with genotype data for HapMap CEU and YRI unrelated individuals. The number of significant principal components was assessed using the minimum average partial test.

*De novo* genotyping for rs73885319 and rs71785313 was performed using the iPLEX Gold assay on the MassArray platform (Sequenom, San Diego, CA). PCR and extension primers were designed using MassArray designer software and publicly available sequence data. Genotyping success rates were 98.1% and 97.6%, respectively. Quality control was assessed by testing for Hardy-Weinberg equilibrium.

## Supporting Information

Figure S1
**Local genetic architectures for the 23 loci.** For each locus, the set of SNPs was bounded by the two SNPs farthest from the query SNP for which 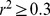 in pairwise comparison with the query SNP in the HapMap CEU data. The length of the associated region was defined as the physical distance between the first two non-associated markers flanking all associated markers. *P*-values are shown based on physical position (NCBI build 36). The light blue curve depicts the recombination rate from the combined HapMap Phase II data. Linkage disequilibrium based on the HUFS sample is color-coded red for *r* ^2^ to the top SNP between 0.8 and 1.0, orange for *r* ^2^ between 0.5 and 0.8, blue for *r* ^2^ between 0.2 and 0.5, and gray for *r* ^2^ between 0 and 0.2. Green arrows indicate the direction of transcription.(PDF)

Figure S2 **Principal components analysis.** Red represents CEU, blue represents YRI, and black represents the HUFS sample. Only the first principal component explained a significant amount of genetic variance.
(PNG)

Figure S3 **Genome-wide Manhattan plots.** (A) Unstratified by ancestry. (B) Stratified by ancestry.
(PNG)

Figure S4 **Genome-wide distribution of admixture proportion.**
(PNG)

Figure S5
**Density plots.** The phenotypic distribution of eGFR was Box-Cox transformed to reduce skew using the transformation 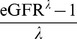 with the maximum likelihood estimate 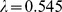. The resulting distribution was winsorized at ±3 SD to reduce kurtosis. These adjustments reduced skew from 2.4 to −0.1 and reduced kurtosis from 25.2 to 1.0. Units for unadjusted glomerular filtration rate are mL/min/1.73 m^2^.(PDF)

Table S1 **Percent variance explained among associated loci.**
(DOC)

Table S2 **Ancestry effects among the associated loci.**
(DOC)

Table S3 **Significant secondary associations.**
(DOC)
