# Supplementary figures and images for: Multiple Loci Associated with Renal Function in African Americans
Source: PLoS One. 2012 Sep 13;7(9):e45112. doi: 10.1371/journal.pone.0045112 (PMC3441677; doi:10.1371/journal.pone.0045112)

SYPL2

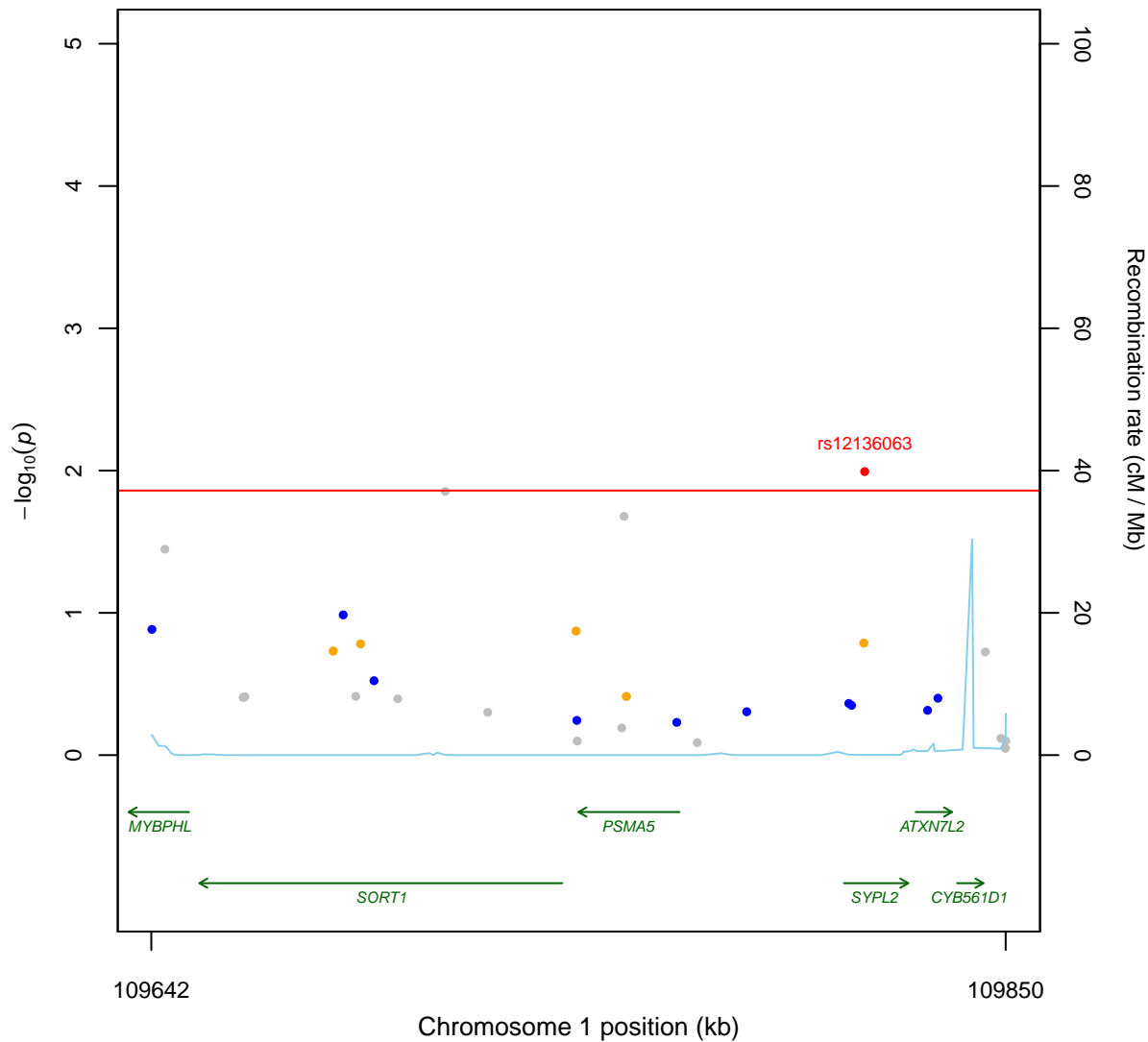

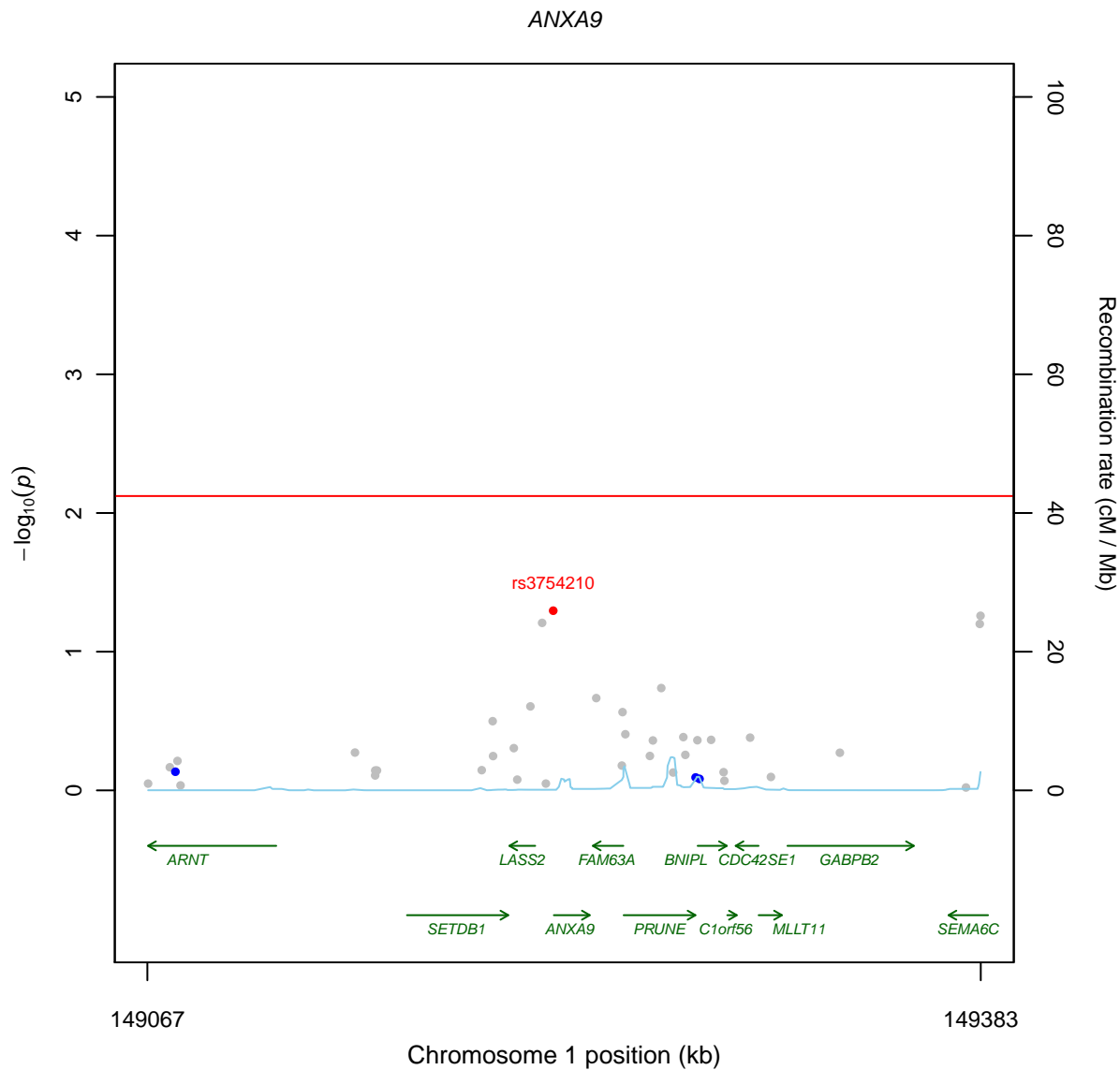

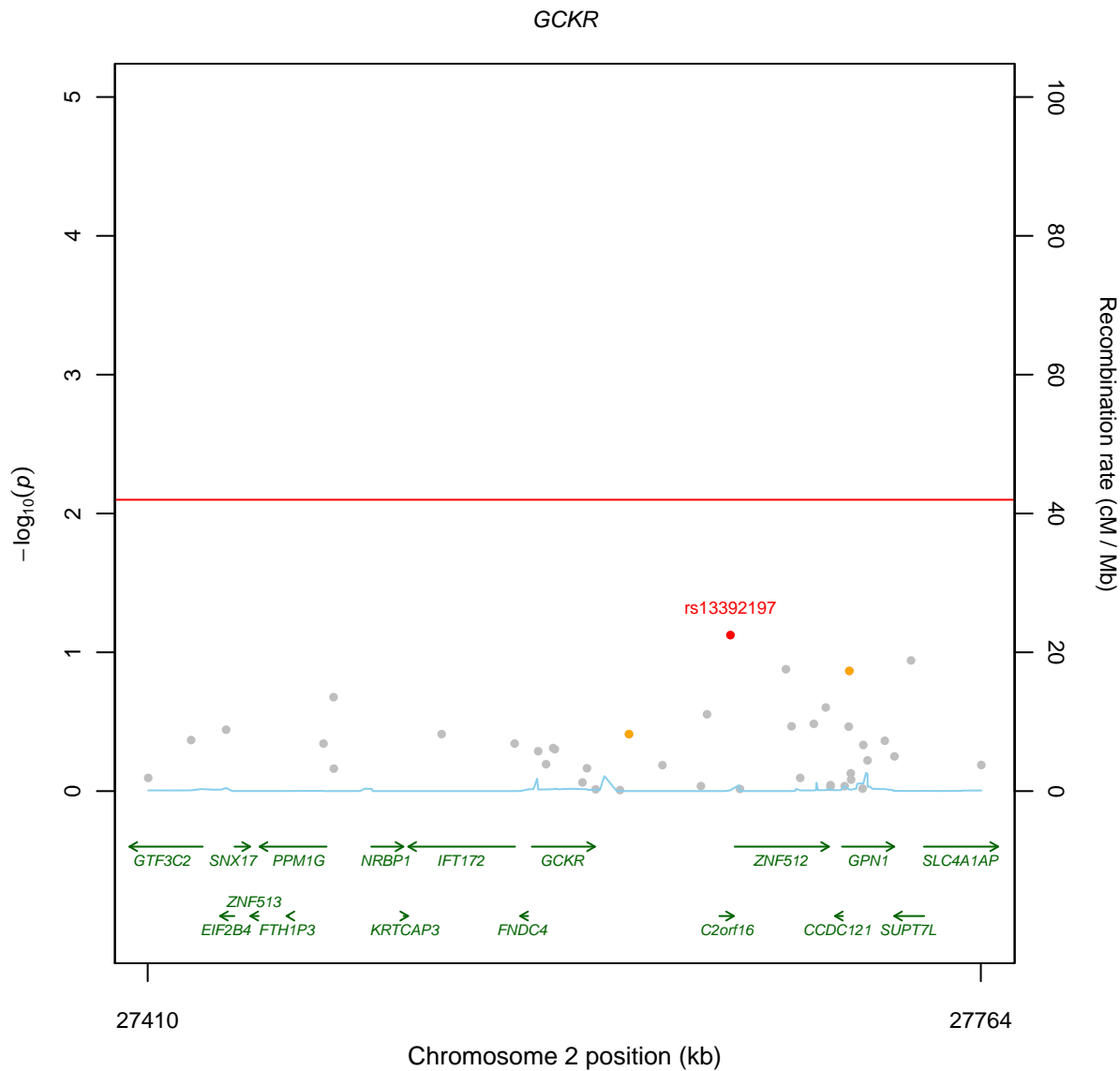

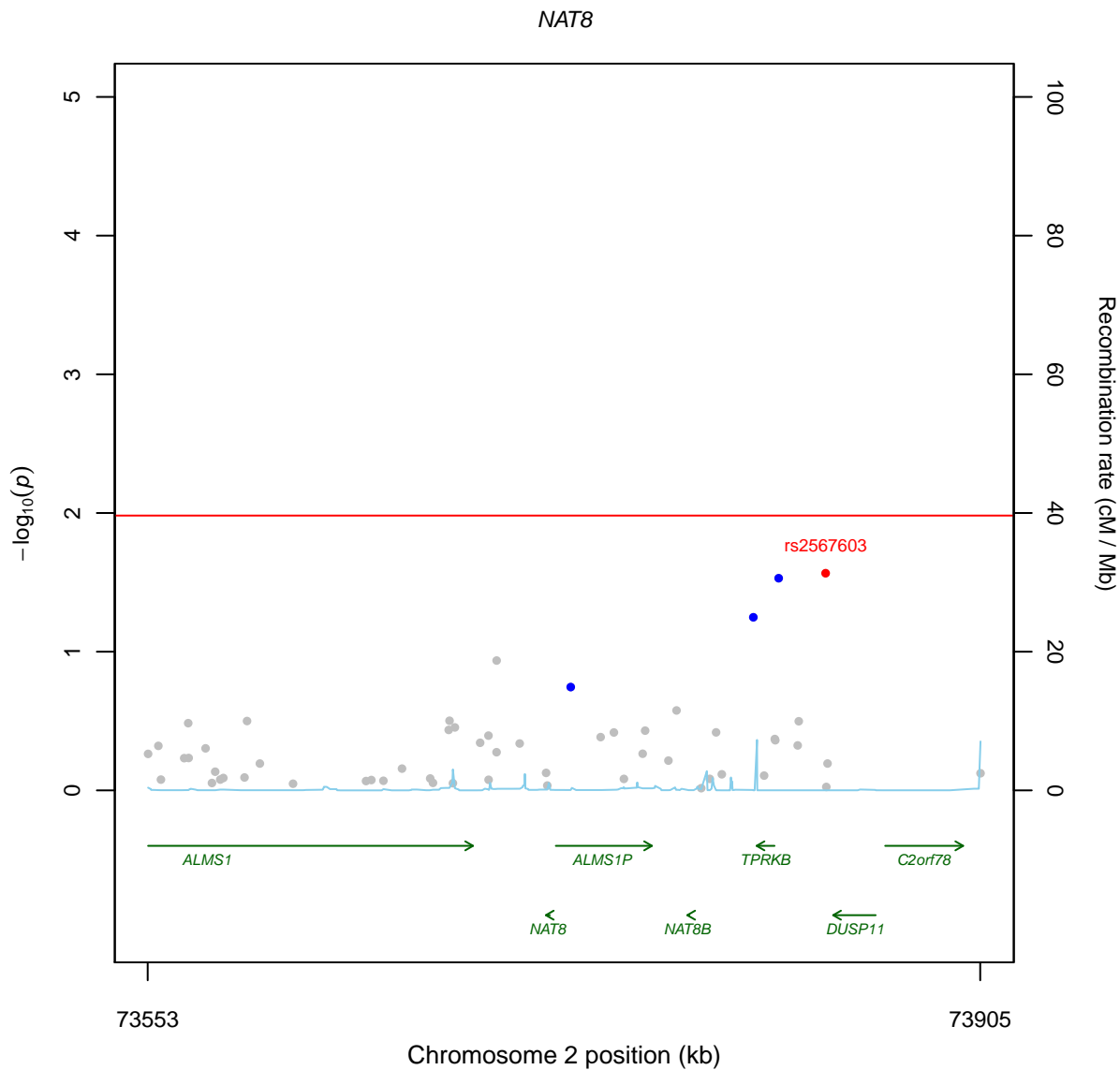

*CPS1*

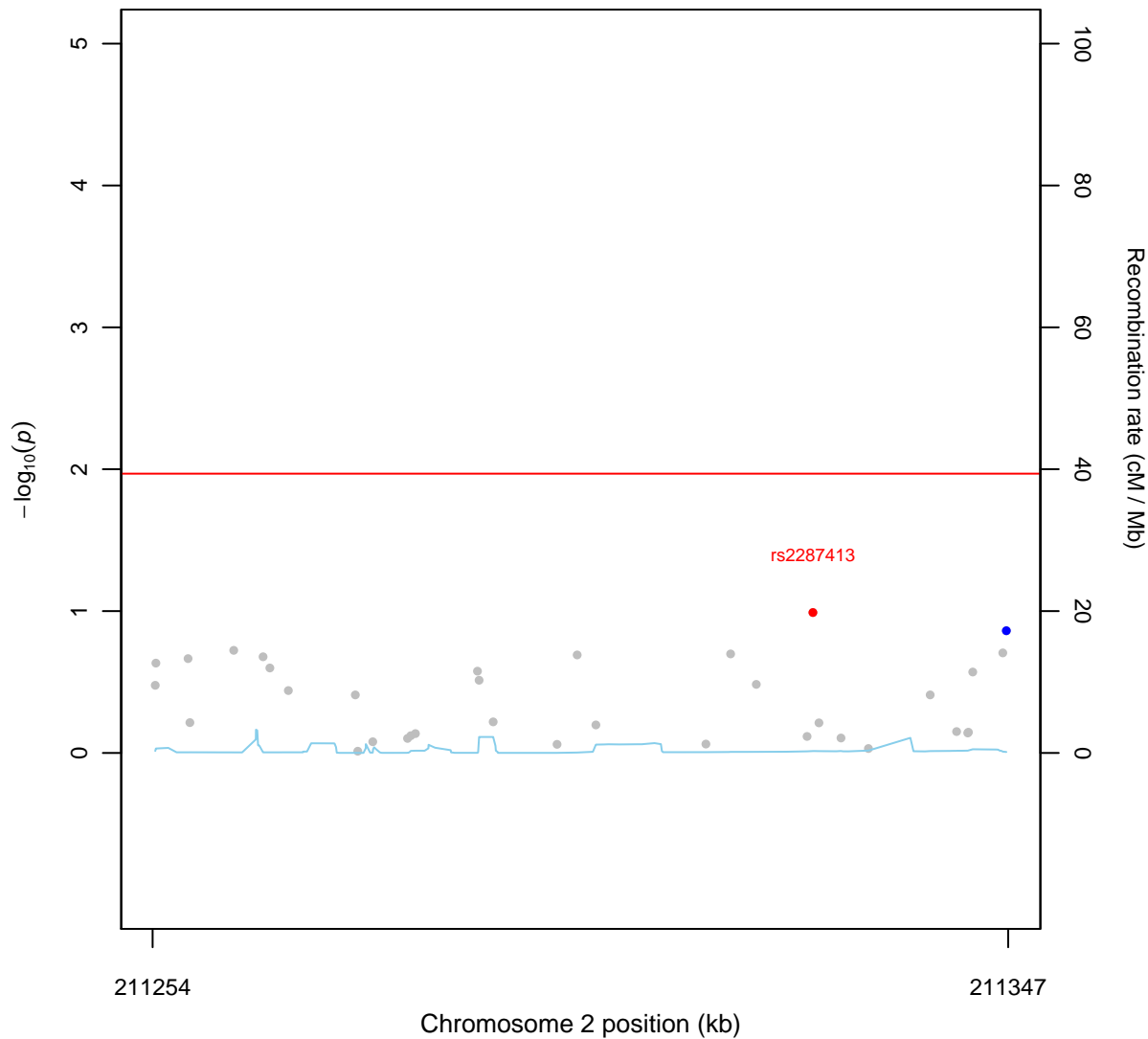

*TFDP2*

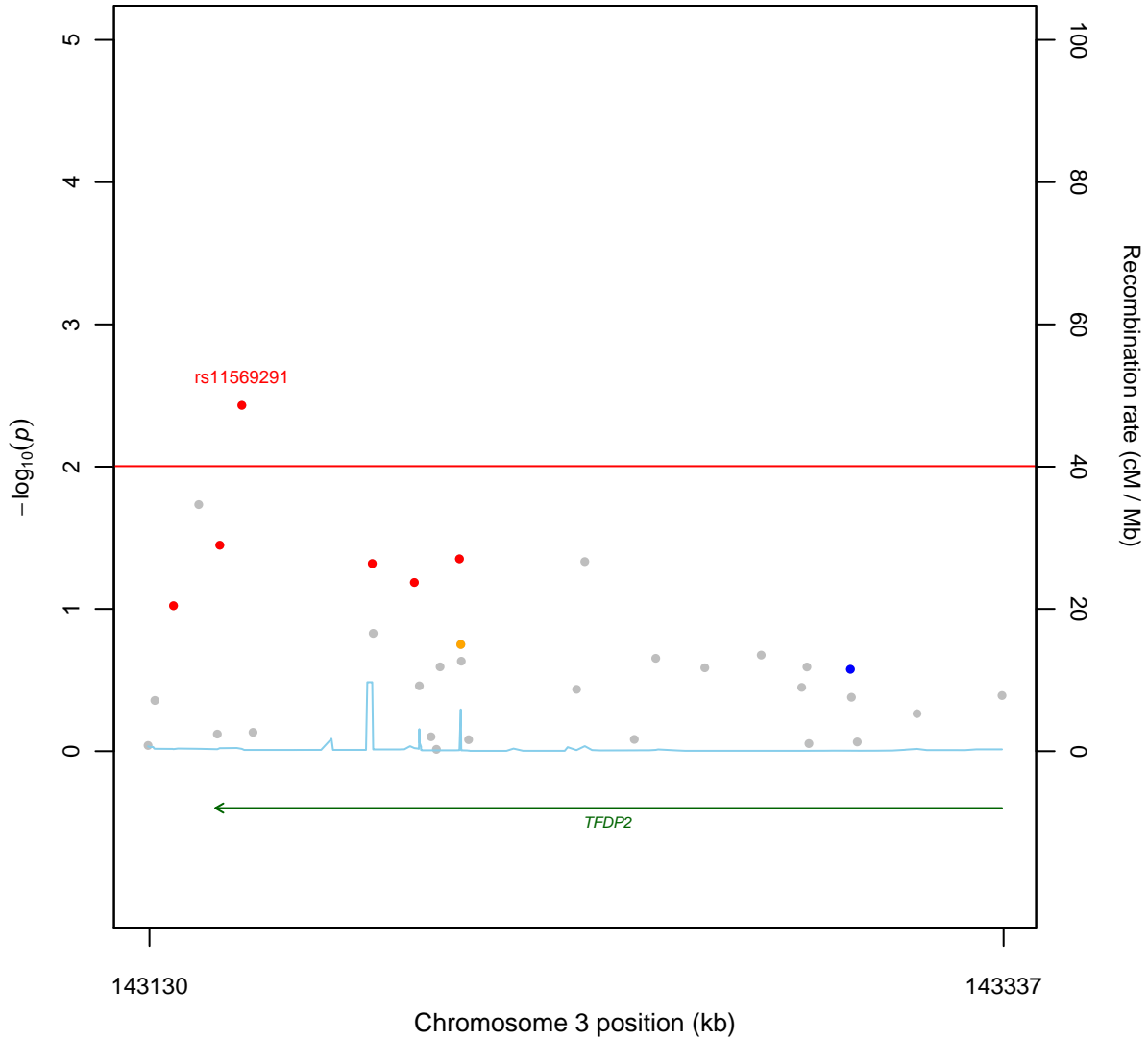

SHROOM3

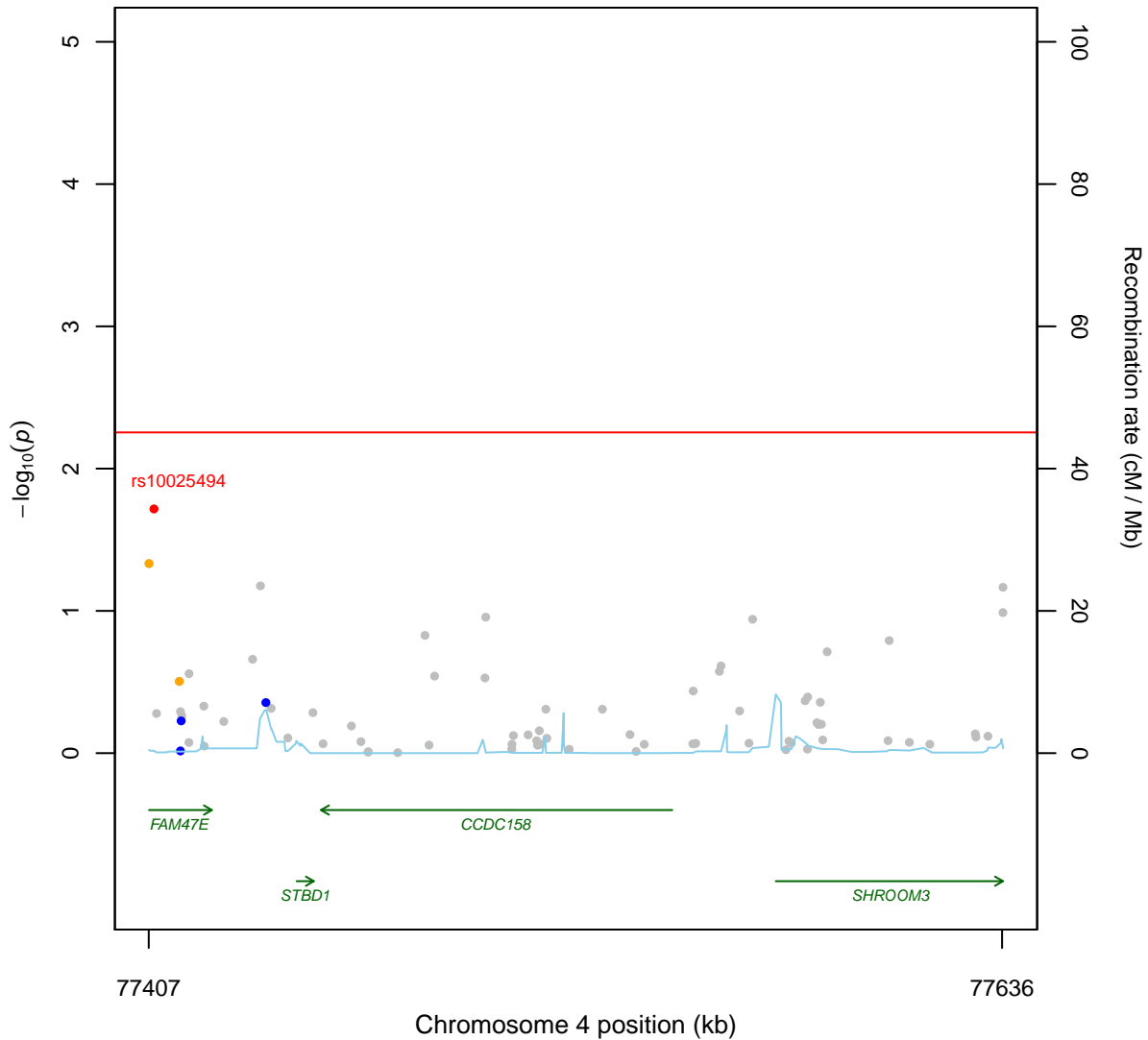

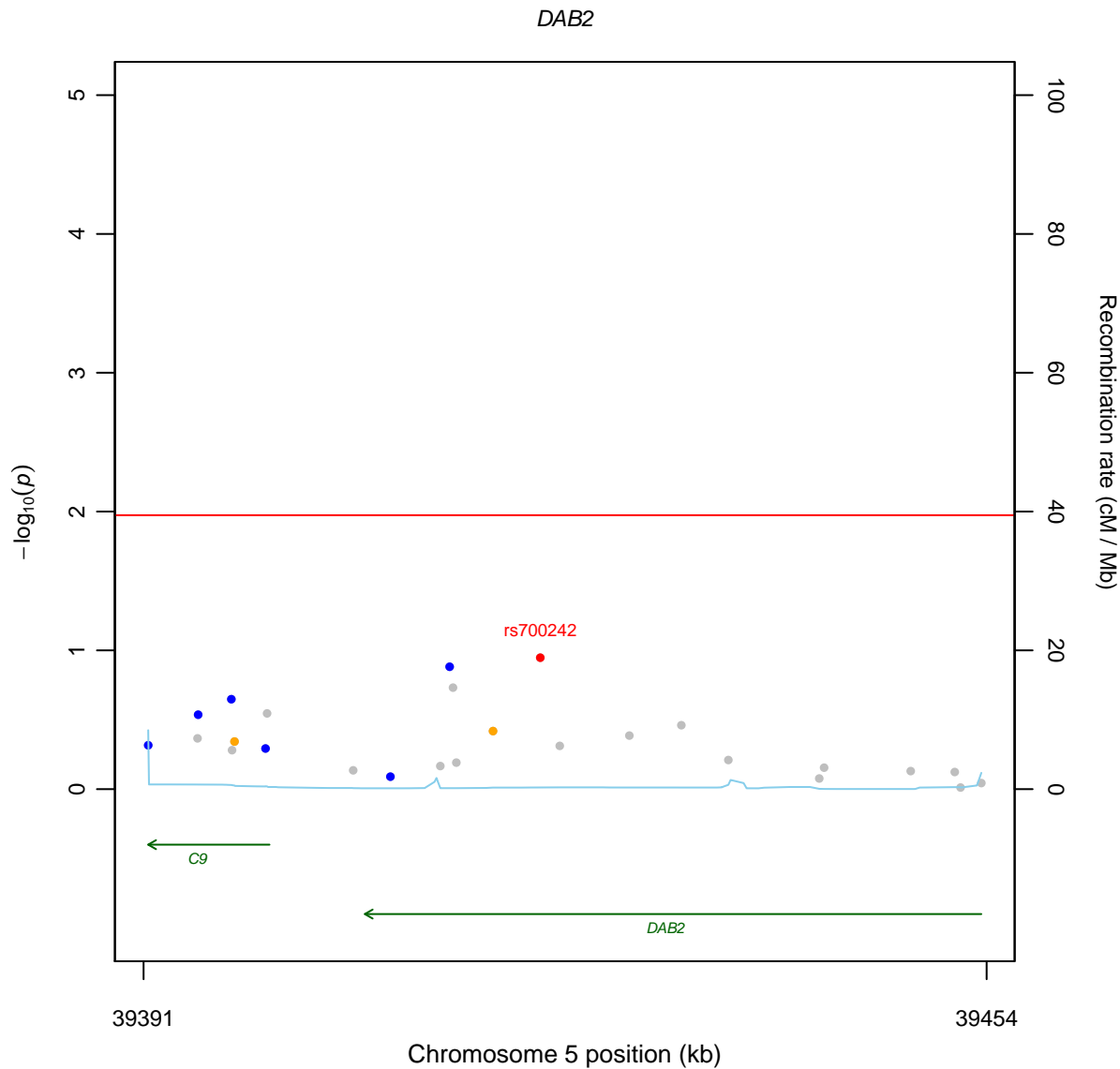

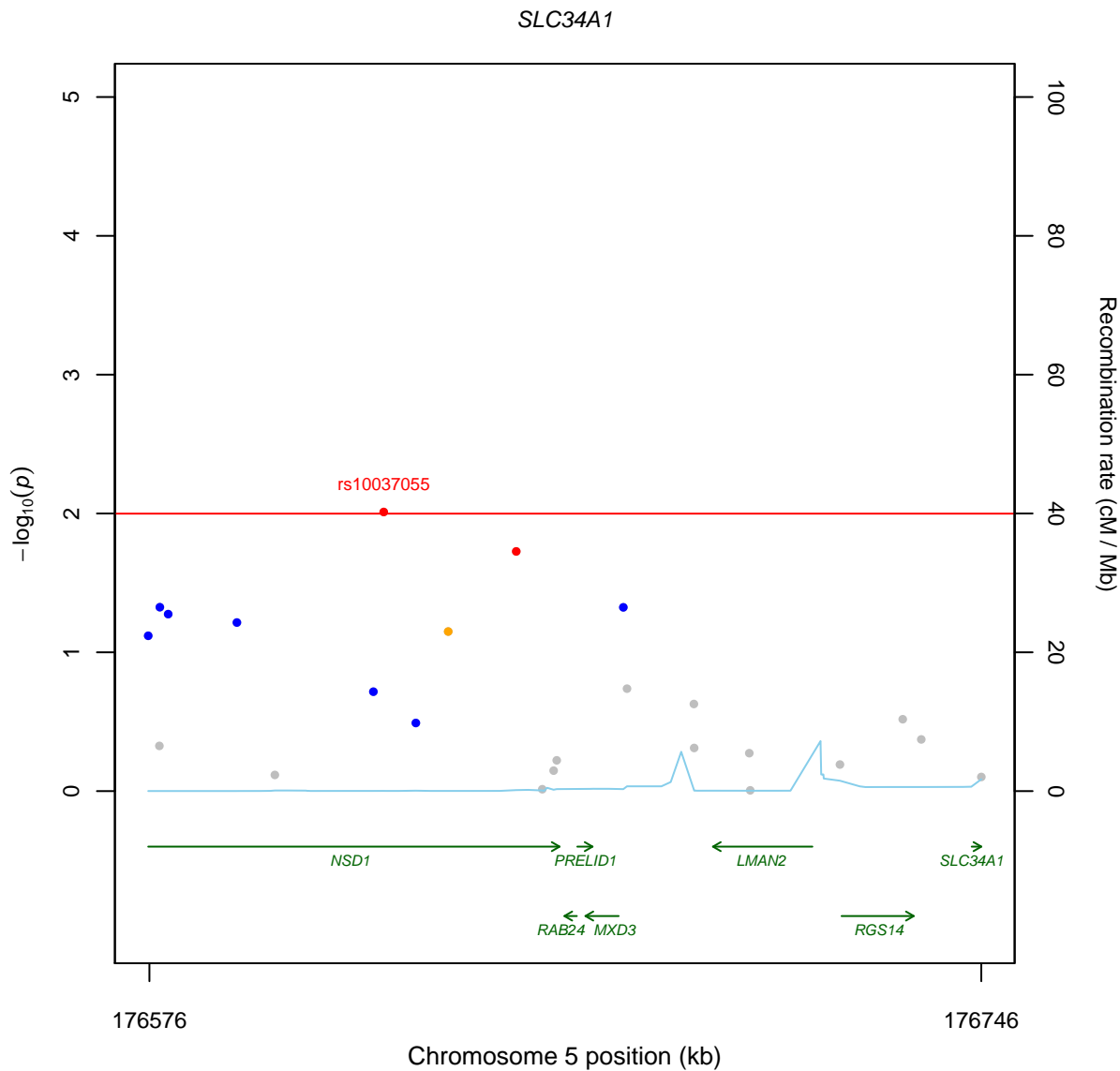

VEGFA

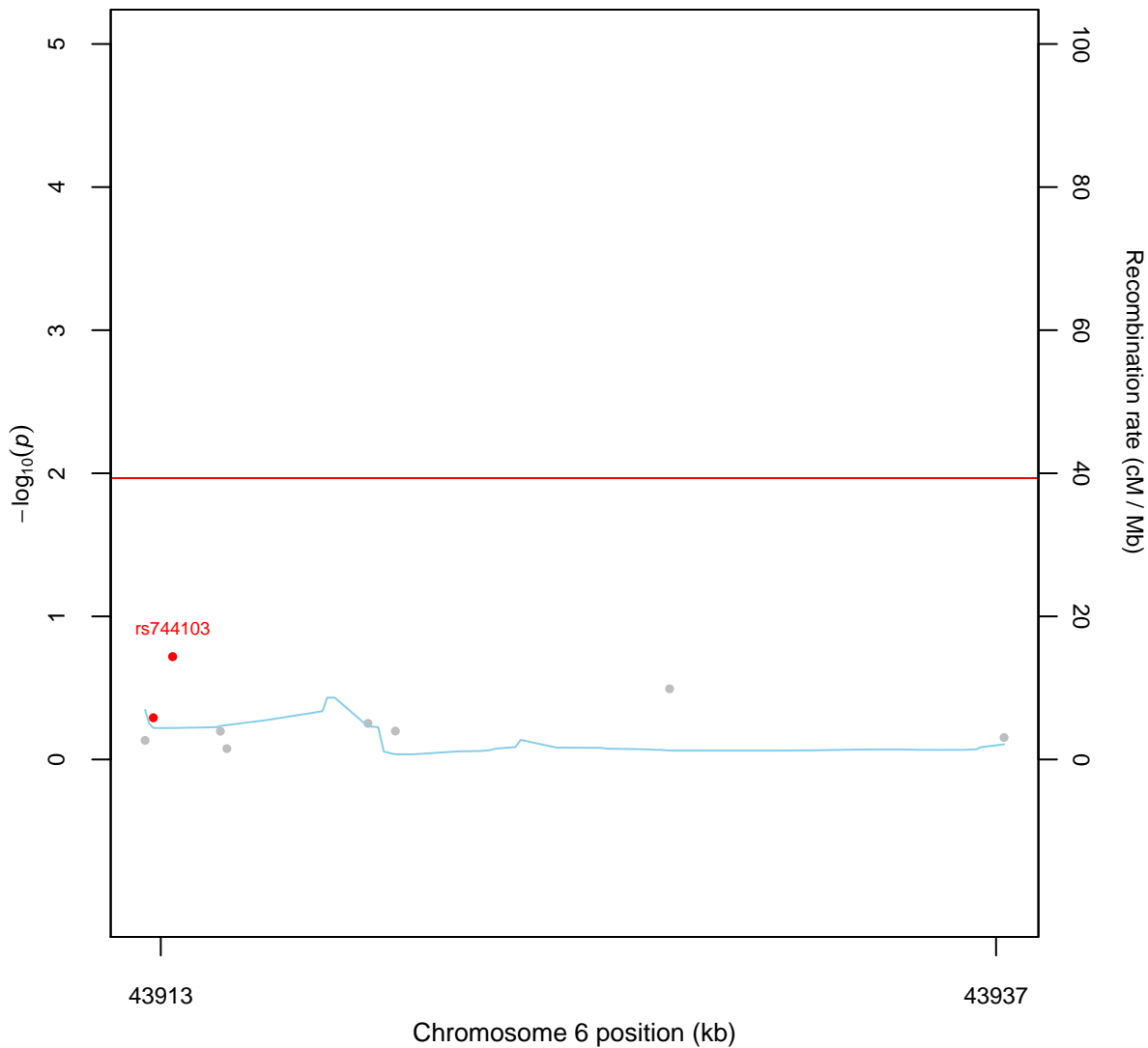

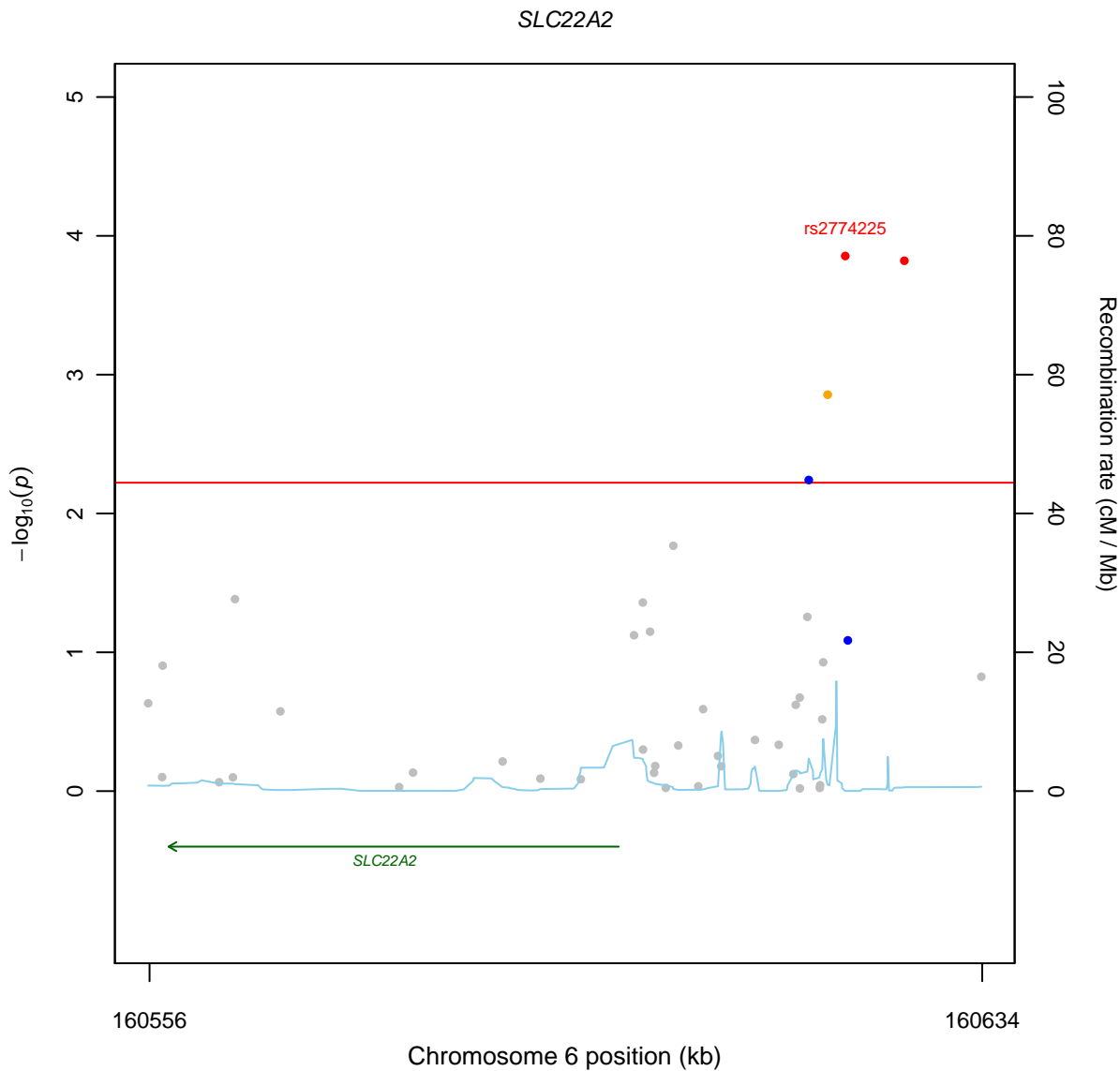

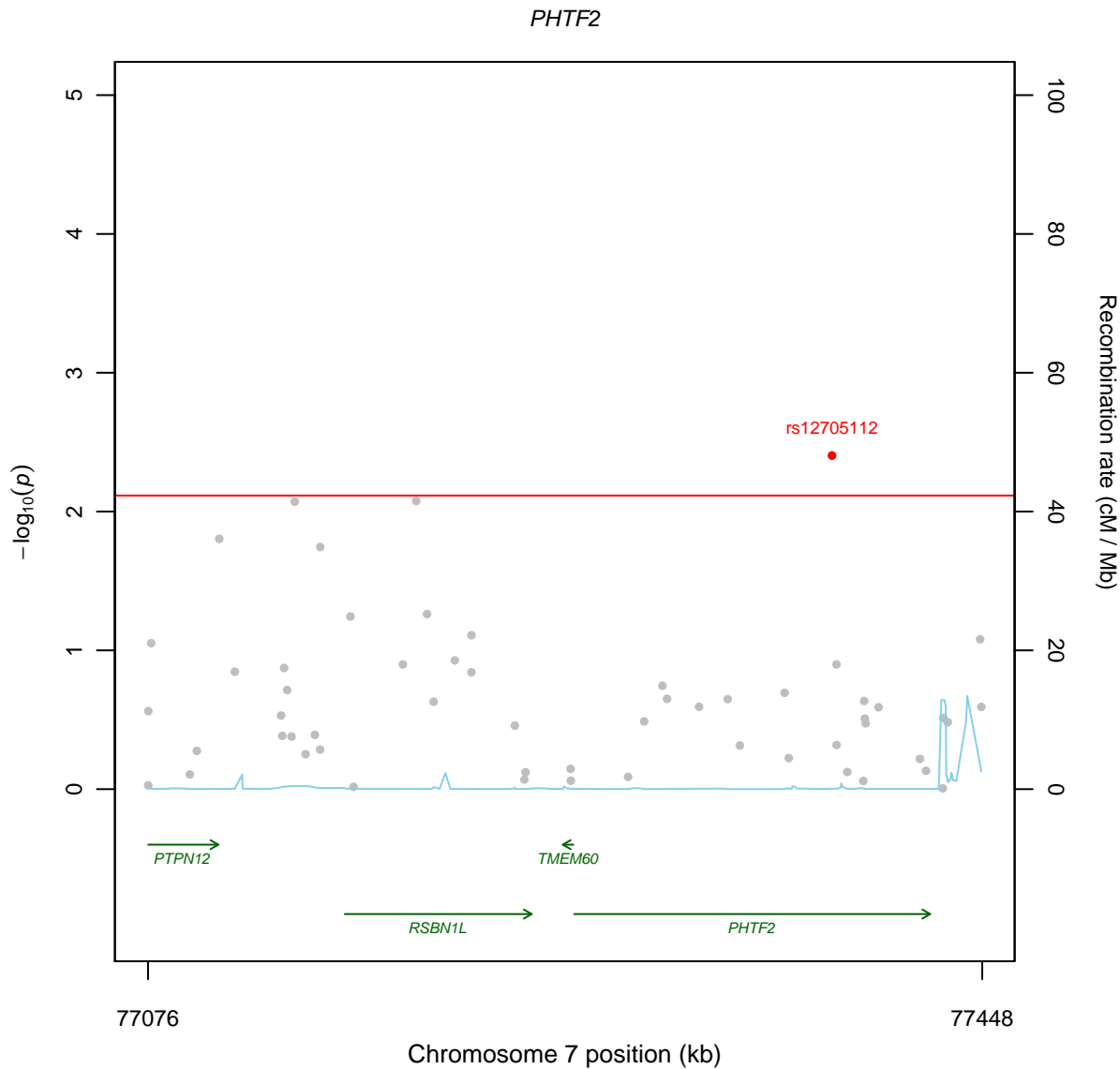

PIP5K1B / FAM122A

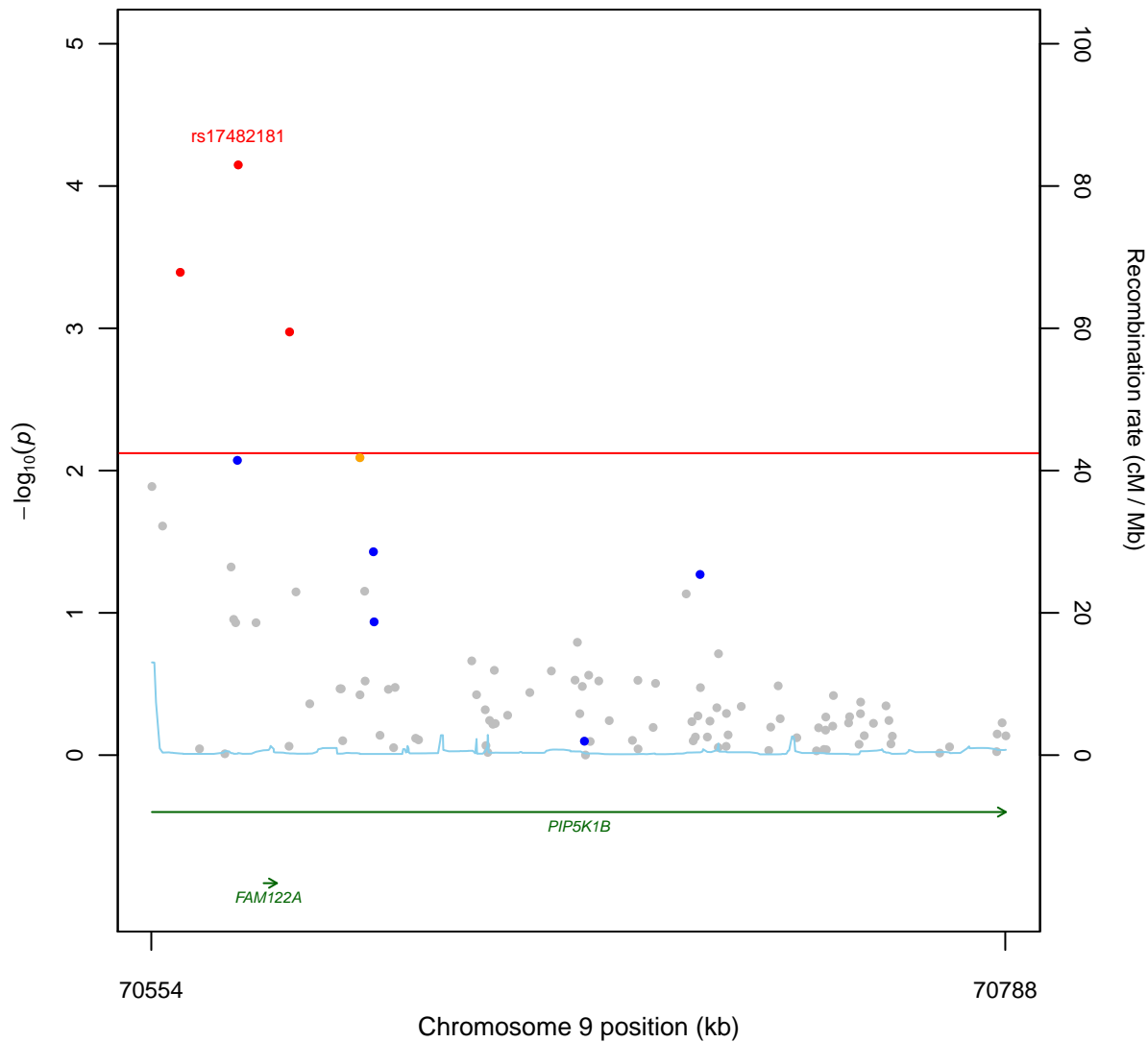

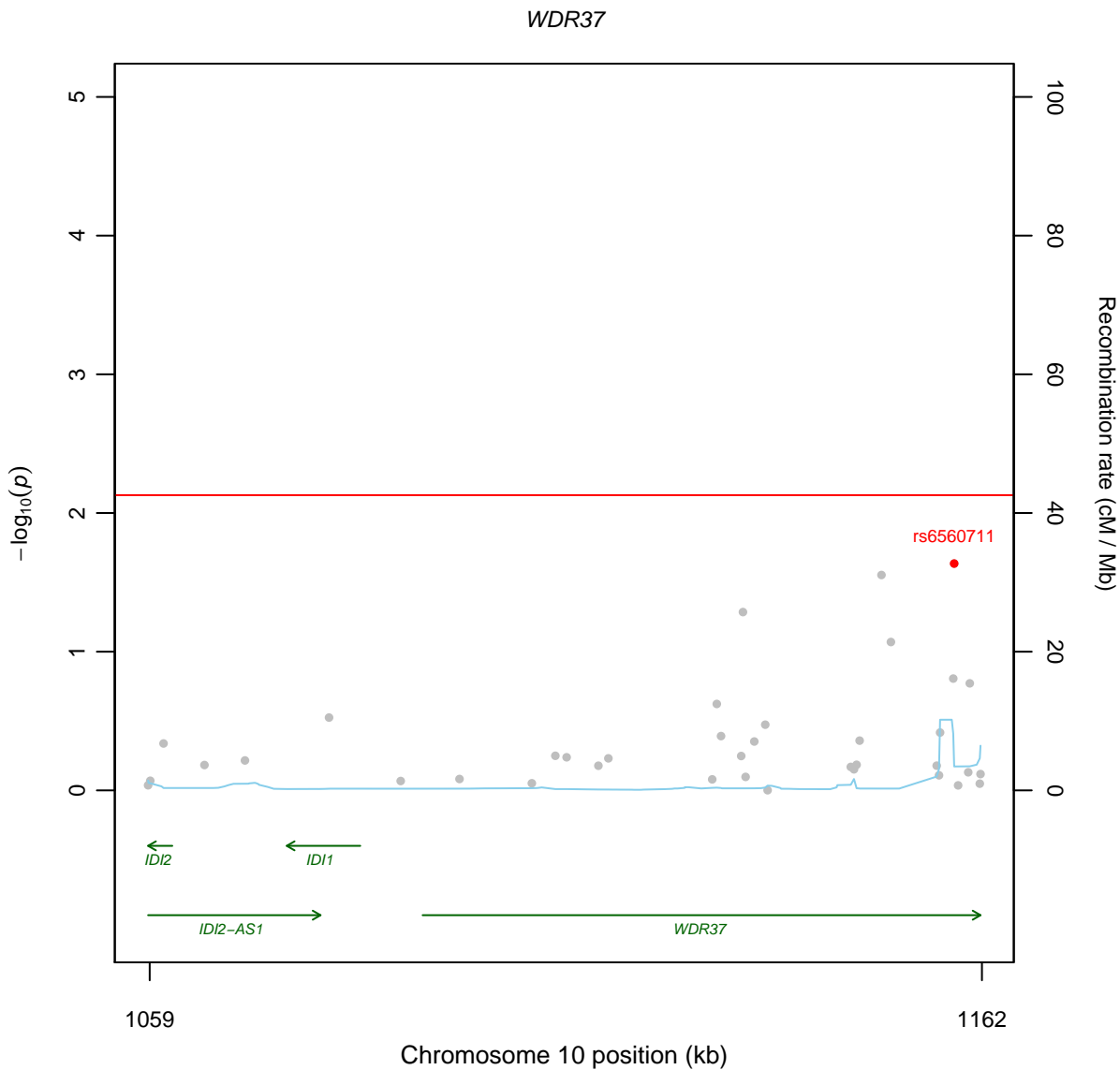

# OVOL1

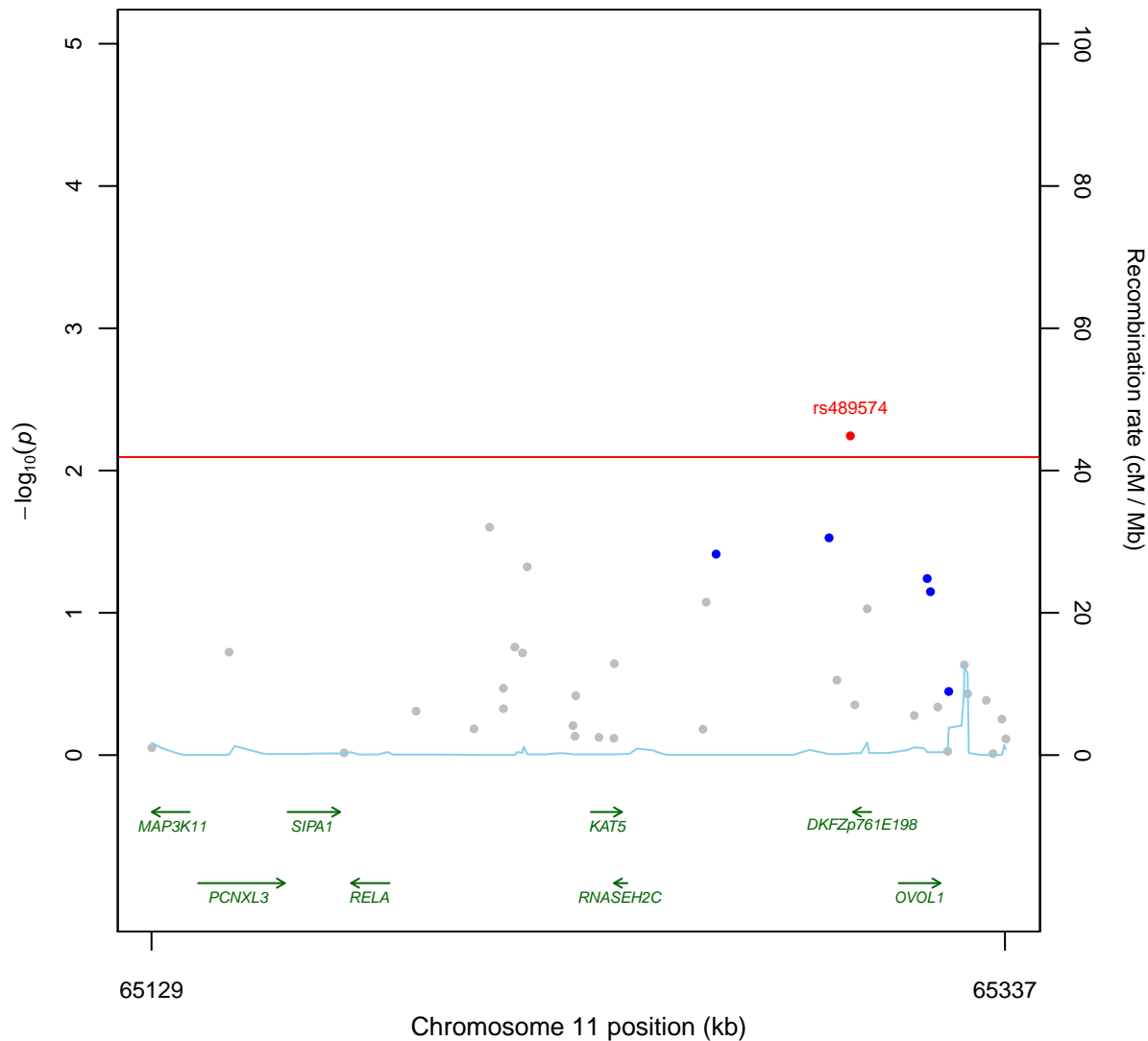

*SLC6A13*

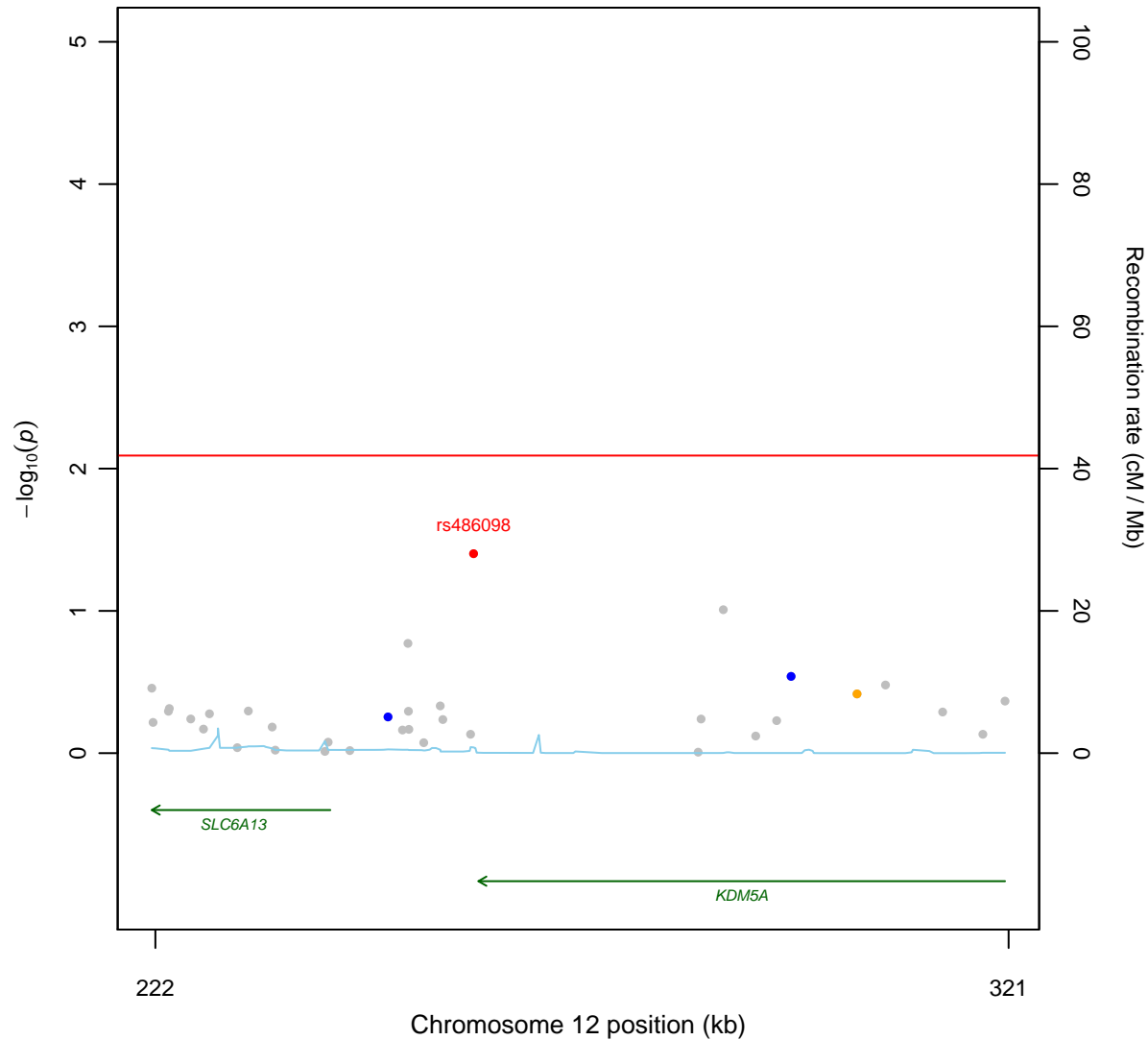

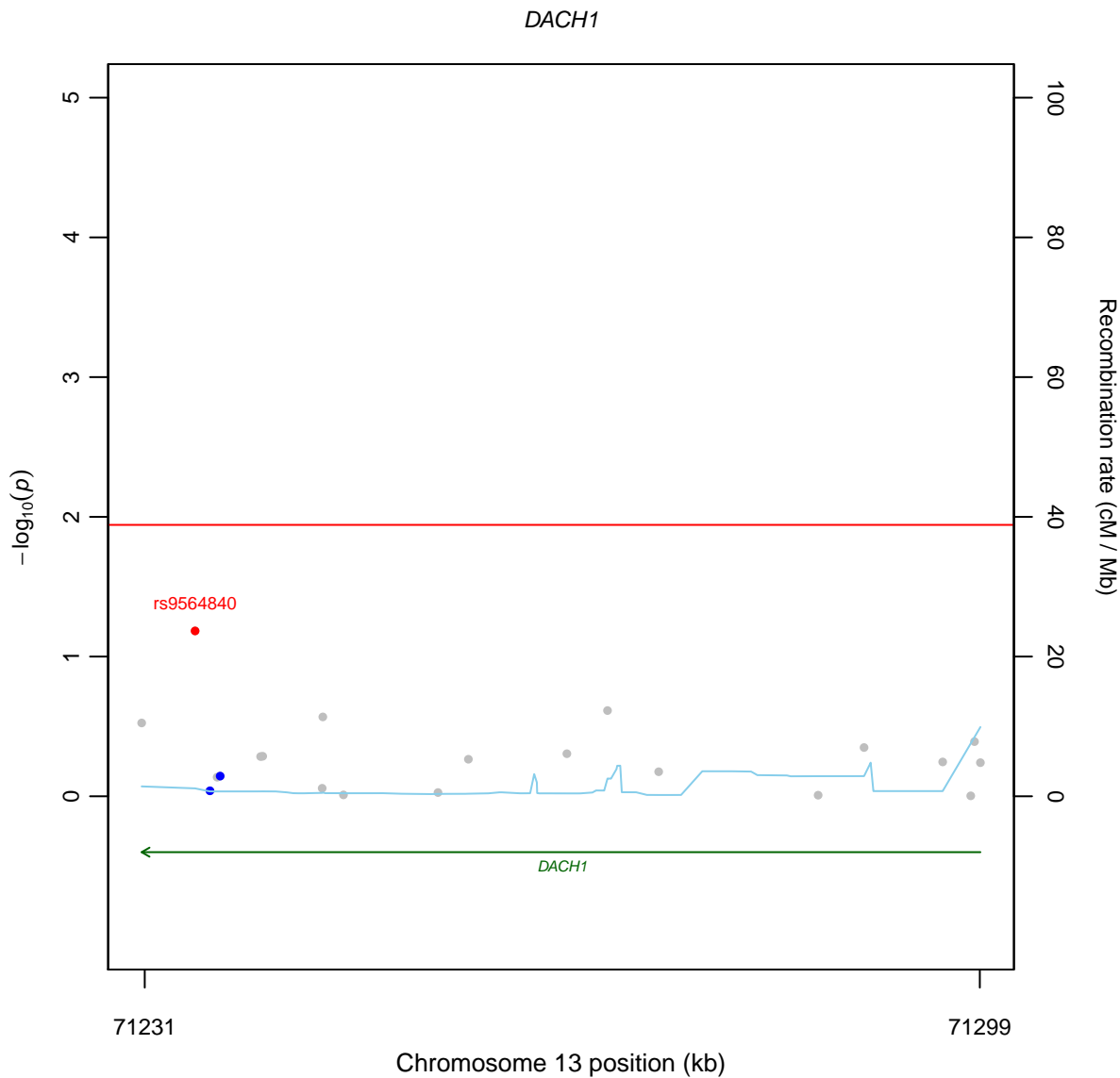

*GATM* / *SPATA5L1*

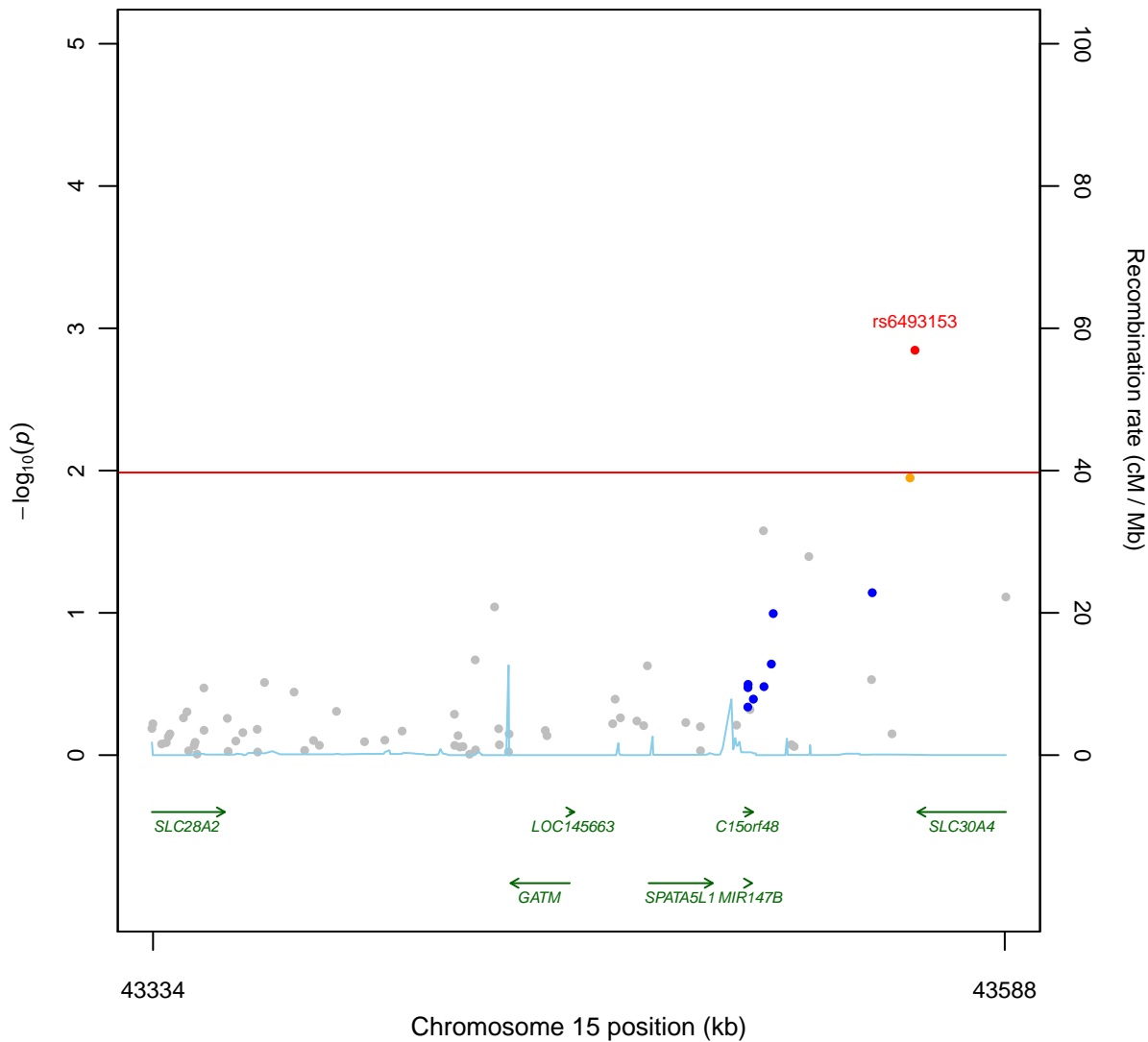

WDR72

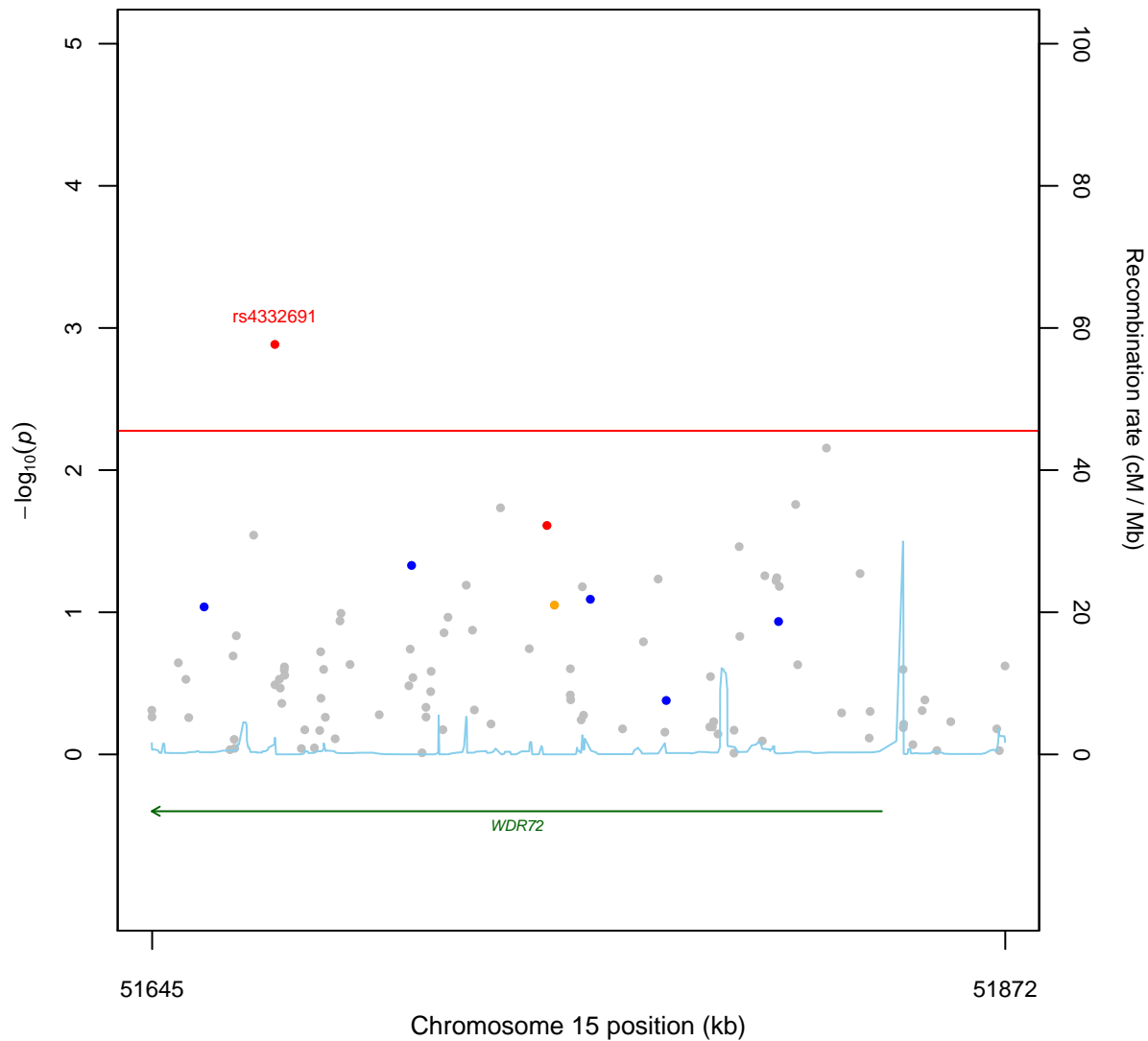

*UBE2Q2*

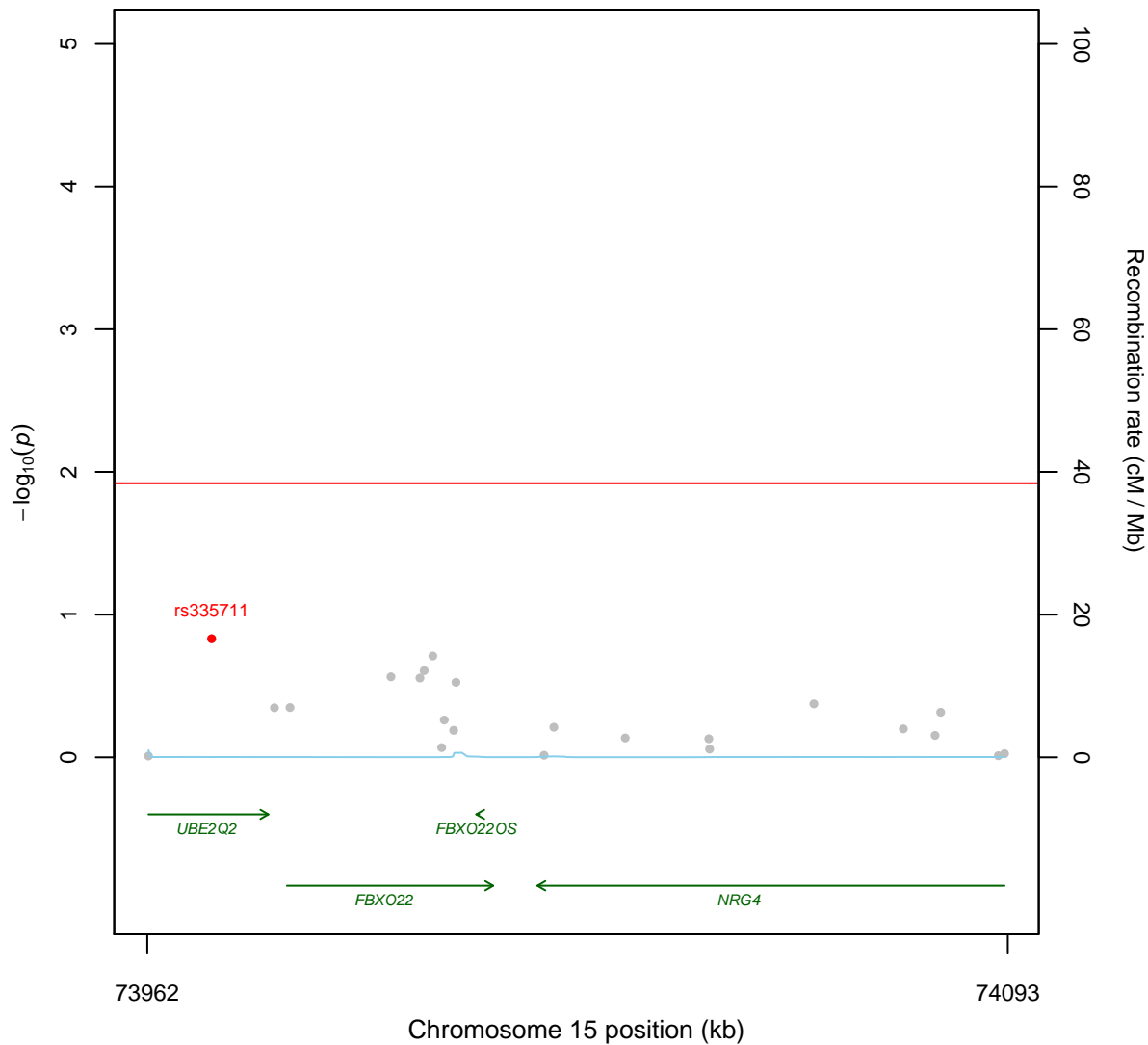

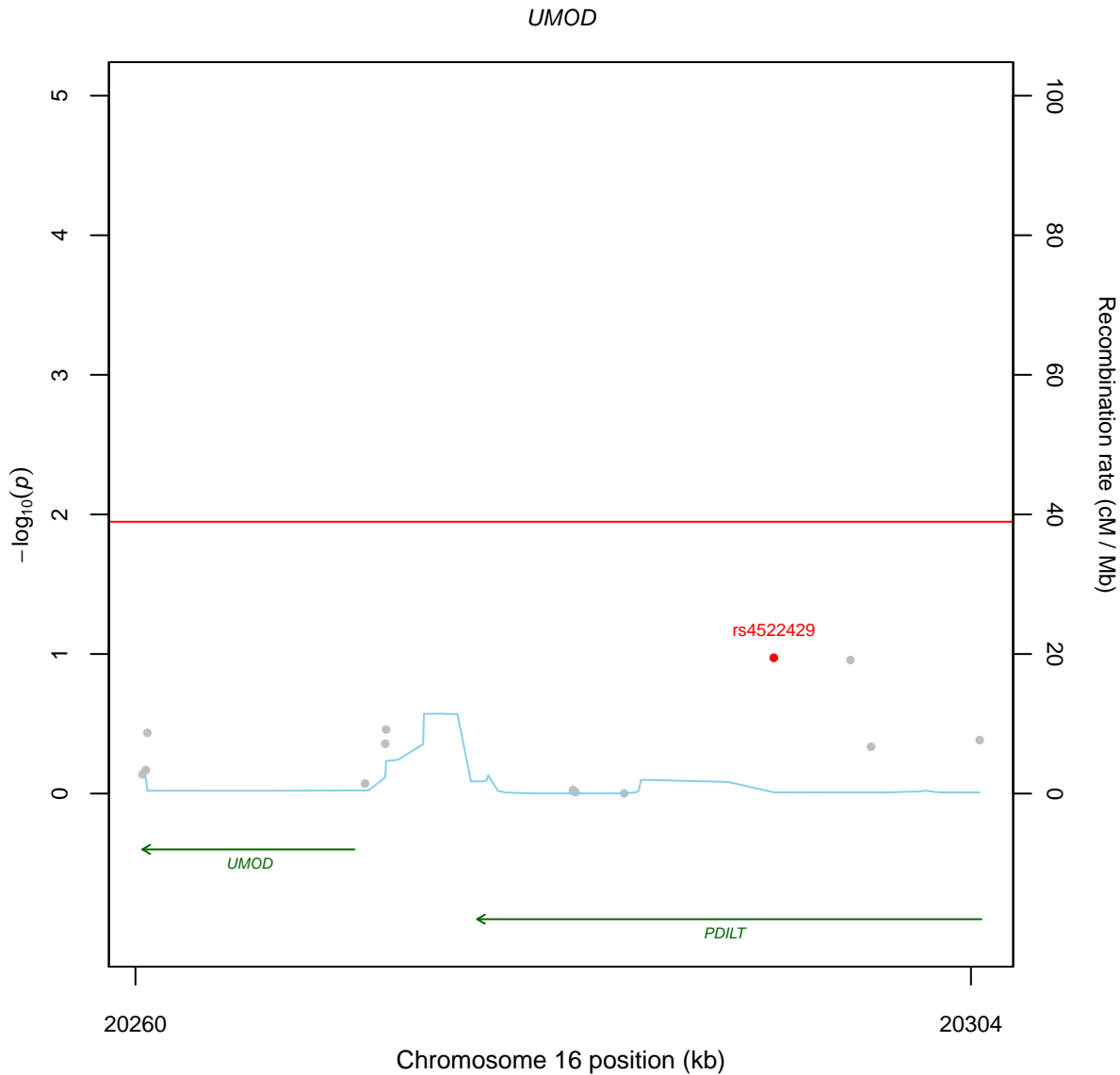

*BCAS3* / *TBX2*

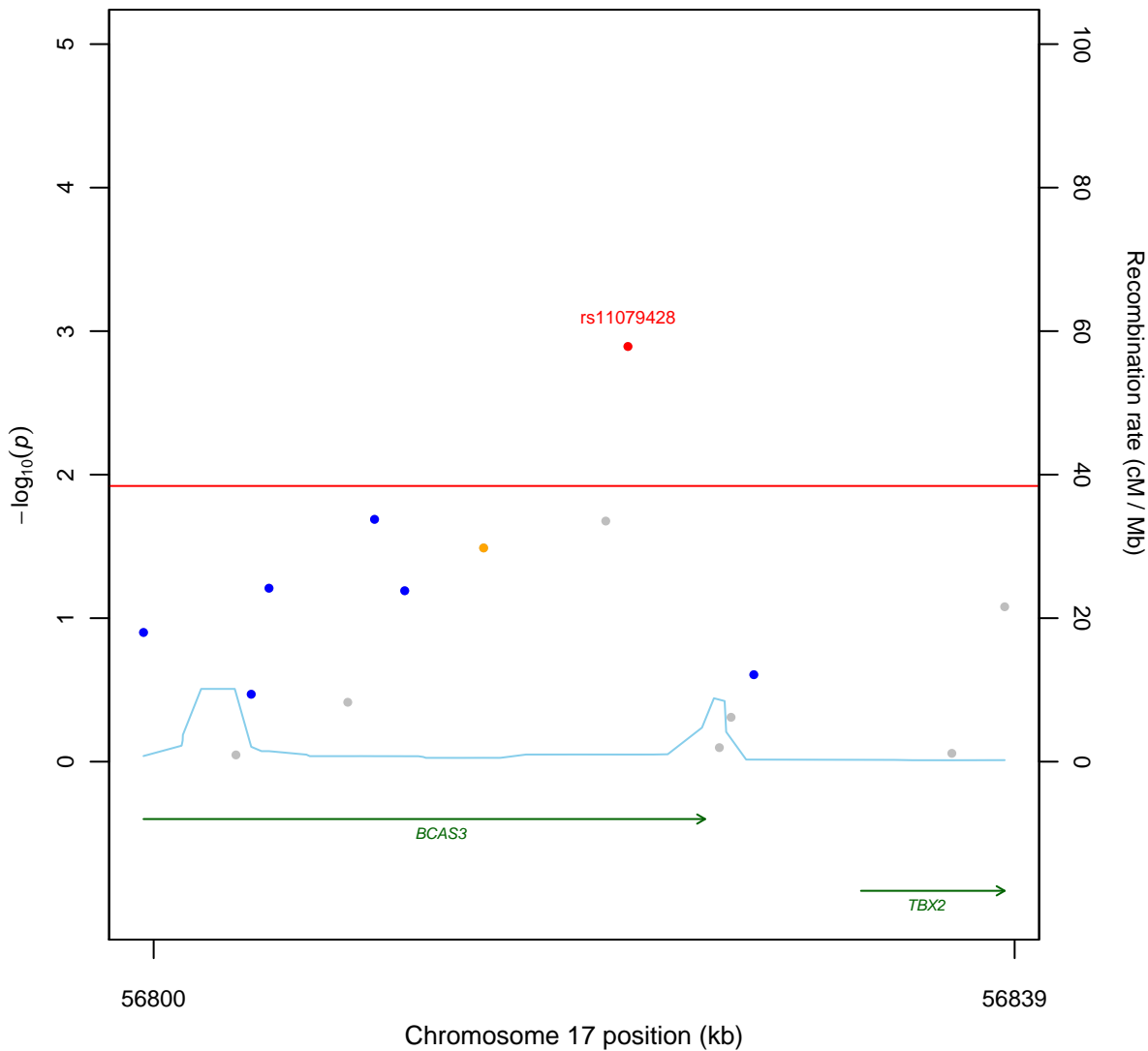

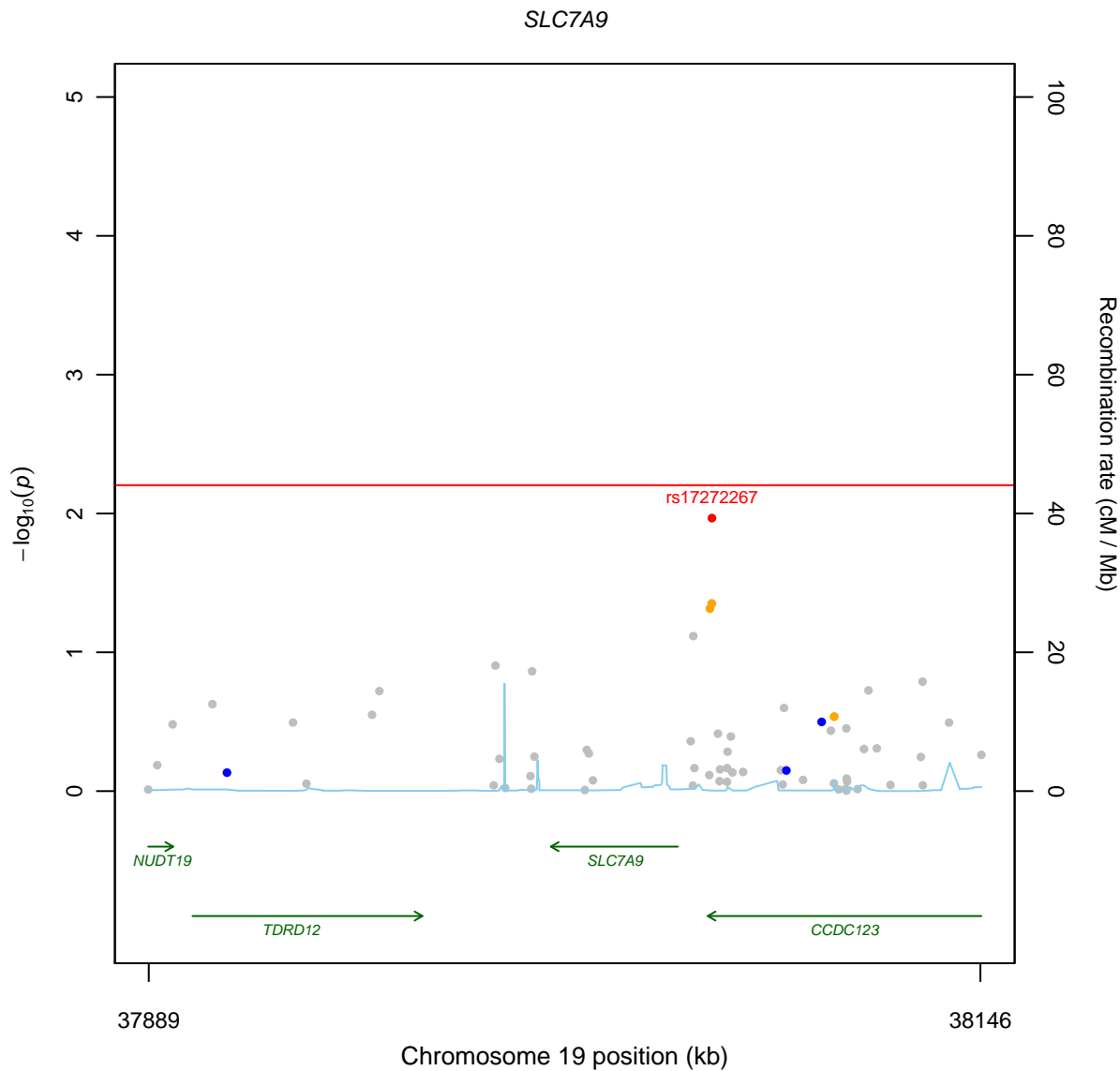

Supplement: Figure S1 — Local genetic architectures for the 23 loci. For each locus, the set of SNPs was bounded by the two SNPs farthest from the query SNP for which in pairwise comparison with the query SNP in the HapMap CEU data. The length of the associated region was defined as the physical distance between the first two non-associated markers flanking all associated markers. P-values are shown based on physical position (NCBI build 36). The light blue curve depicts the recombination rate from the combined HapMap Phase II data. Linkage disequilibrium based on the HUFS sample is color-coded red for r 2 to the top SNP between 0.8 and 1.0, orange for r 2 between 0.5 and 0.8, blue for r 2 between 0.2 and 0.5, and gray for r 2 between 0 and 0.2. Green arrows indicate the direction of transcription. (PDF) [file pone.0045112.s001.pdf]

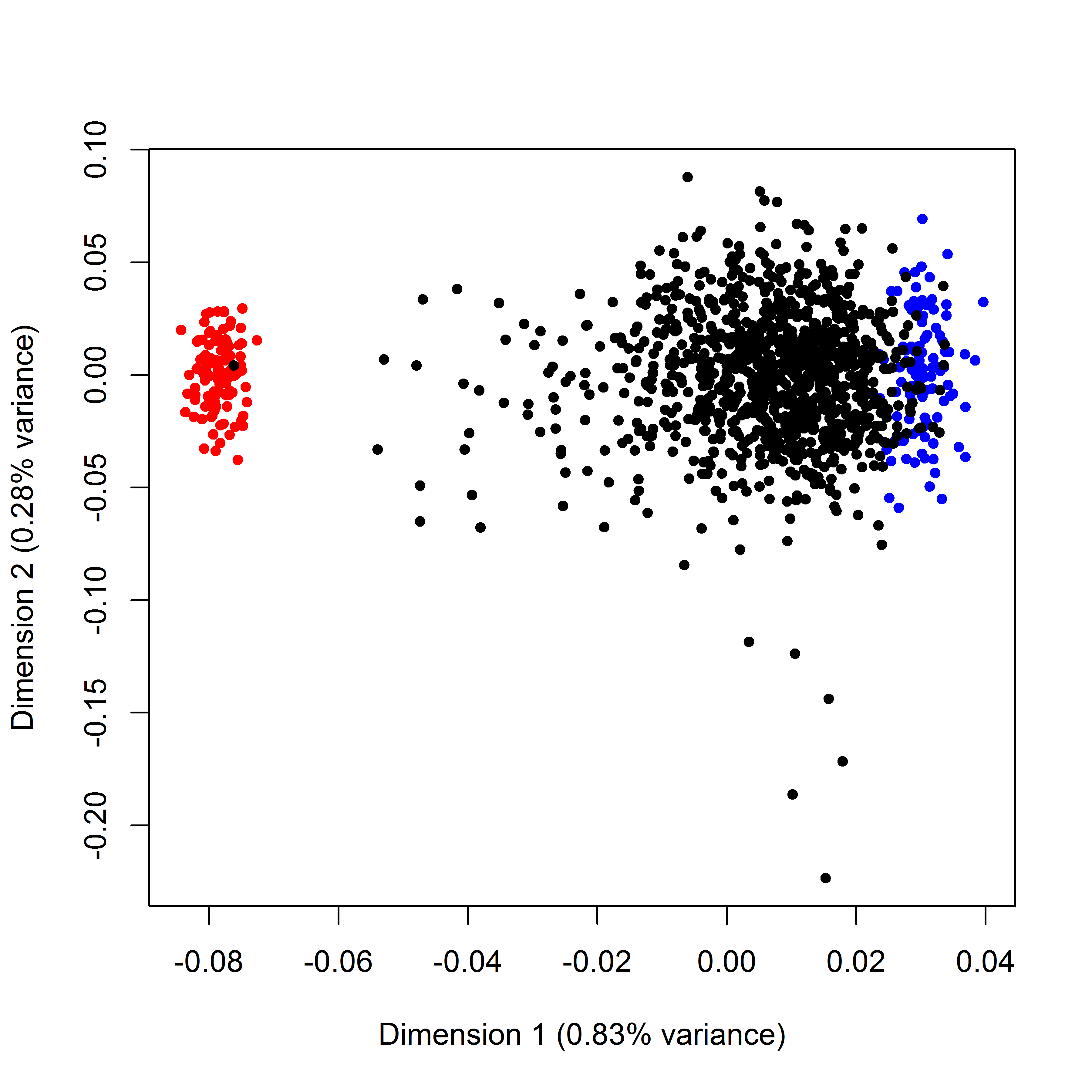

Supplement: Figure S2 — Principal components analysis. Red represents CEU, blue represents YRI, and black represents the HUFS sample. Only the first principal component explained a significant amount of genetic variance. (PNG) [file pone.0045112.s002.png]

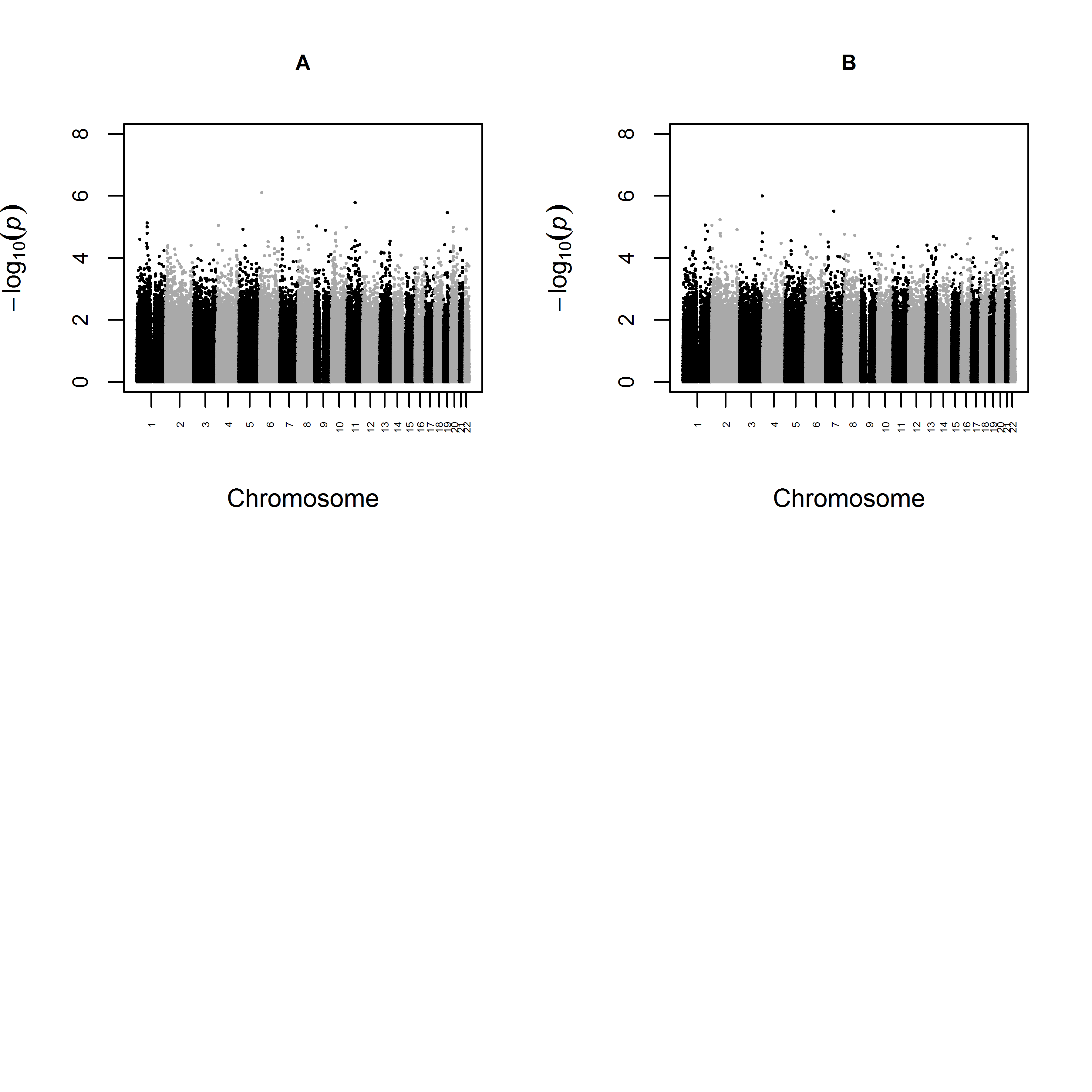

Supplement: Figure S3 — Genome-wide Manhattan plots. (A) Unstratified by ancestry. (B) Stratified by ancestry. (PNG) [file pone.0045112.s003.png]

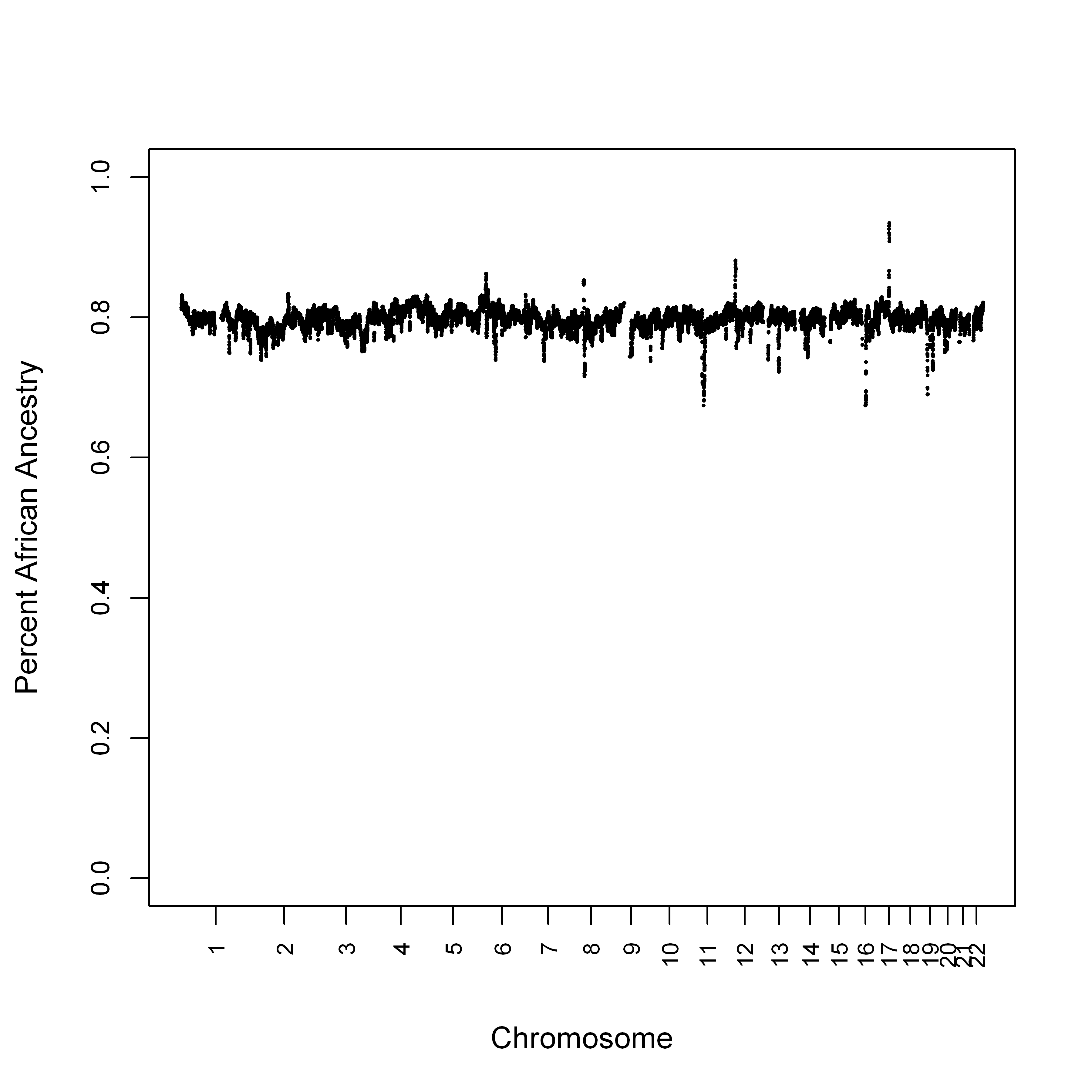

Supplement: Figure S4 — Genome-wide distribution of admixture proportion. (PNG) [file pone.0045112.s004.png]

**A**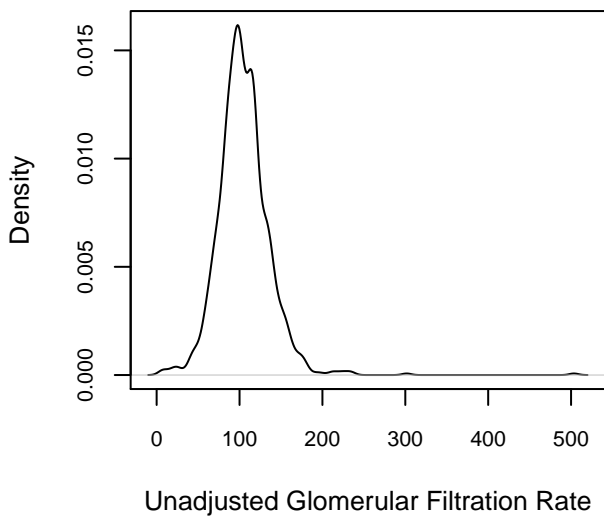**B**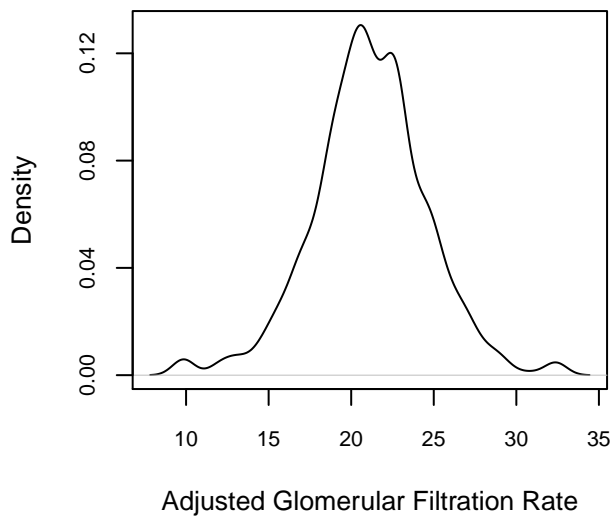

Supplement: Figure S5 — Density plots. The phenotypic distribution of eGFR was Box-Cox transformed to reduce skew using the transformation with the maximum likelihood estimate . The resulting distribution was winsorized at ±3 SD to reduce kurtosis. These adjustments reduced skew from 2.4 to −0.1 and reduced kurtosis from 25.2 to 1.0. Units for unadjusted glomerular filtration rate are mL/min/1.73 m2. (PDF) [file pone.0045112.s005.pdf]
