# Supplementary material for: Multiple Loci Associated with Renal Function in African Americans
Source: PLoS One. 2012 Sep 13;7(9):e45112. doi: 10.1371/journal.pone.0045112 (PMC3441677; doi:10.1371/journal.pone.0045112)
Supplement: Table S1 — Percent variance explained among associated loci. (DOC) [file pone.0045112.s006.doc]

**Supplementary Table S1. Percent variance explained among associated** loci.

| **Chr** | **Gene** | **Best Marker** | **Position (bp)** | **Coded Allele** | **Allele Frequency** | **β_meta_** | **SE_meta_** | **R^2^** |
| --- | --- | --- | --- | --- | --- | --- | --- | --- |
| 1 | *SYPL2* | rs12136063 | 109815693 | G | 0.683 | 0.426 | 0.166 | 0.0064 |
| 3 | *TFDP2* | rs11569291 | 143152375 | A | 0.981 | -1.670 | 0.575 | 0.0085 |
| 5 | *SLC34A1* | rs10037055 | 176623885 | G | 0.515 | 0.381 | 0.147 | 0.0059 |
| 6 | *SLC22A2* | rs2774225 | 160621171 | G | 0.672 | 0.559 | 0.147 | 0.0113 |
| 7 | *PHTF2* | rs12705112 | 77380997 | C | 0.982 | -1.473 | 0.511 | 0.0063 |
| 9 | *PIP5K1B/FAM122A* | rs17482181 | 70577820 | C | 0.800 | 0.705 | 0.178 | 0.0130 |
| 11 | *OVOL1* | rs489574 | 65299315 | G | 0.777 | 0.469 | 0.170 | 0.0062 |
| 15 | *SLC30A4* | rs6493153 | 43561217 | A | 0.716 | -0.516 | 0.162 | 0.0089 |
| 15 | *WDR72* | rs4332691 | 51677654 | C | 0.908 | 0.929 | 0.289 | 0.0118 |
| 17 | *BCAS3/TBX2* | rs11079428 | 56821483 | T | 0.691 | 0.535 | 0.166 | 0.0100 |
| 22 | *APOL1* | rs71785313 | 36662046-36662051 | DEL | 0.122 | -0.419 | 0.210 | 0.0031 |
