# Supplementary material for: Multiple Loci Associated with Renal Function in African Americans
Source: PLoS One. 2012 Sep 13;7(9):e45112. doi: 10.1371/journal.pone.0045112 (PMC3441677; doi:10.1371/journal.pone.0045112)
Supplement: Table S2 — Ancestry effects among the associated loci. (DOC) [file pone.0045112.s007.doc]

**Supplementary Table S2. Ancestry effects among the associat**ed loci.

| **Chr** | **Marker** | **Coded Allele** | **Allele Frequency** | ***n*_0_** | ***n*_2_** | **AF_0_** | **AF_2_** | **Test of Proportions *P* ^a^** | **β_0_** | **SE_0_** | **β_2_** | **SE_2_** | **Test of Effect Homogeneity *P* ^b^** | **Admixture *P* ^c^** |
| --- | --- | --- | --- | --- | --- | --- | --- | --- | --- | --- | --- | --- | --- | --- |
| 1 | rs12136063 | G | 0.683 | 56 | 663 | 0.250 | 0.790 | 6.59×10^-36^ | -0.310 | 0.581 | 0.546 | 0.214 | 0.167 | 0.494 |
| 3 | rs11569291 | A | 0.981 | 61 | 642 | 0.869 | 1 | 1.99×10^-36^ | -1.058 | 0.986 | NA | NA | NA | 0.868 |
| 5 | rs10037055 | G | 0.515 | 48 | 661 | 0.823 | 0.446 | 2.04×10^-12^ | 0.733 | 1.129 | 0.388 | 0.172 | 0.762 | 0.123 |
| 6 | rs2774225 | G | 0.672 | 54 | 666 | 0.713 | 0.656 | 0.274 | 0.464 | 0.893 | 0.484 | 0.179 | 0.983 | 0.361 |
| 7 | rs12705112 | C | 0.982 | 46 | 646 | 0.902 | 0.999 | 1.82×10^-23^ | -1.192 | 0.912 | -1.371 | 3.216 | 0.957 | 0.294 |
| 9 | rs17482181 | C | 0.800 | 81 | 593 | 0.722 | 0.827 | 1.84×10^-3^ | 0.798 | 0.551 | 0.594 | 0.240 | 0.734 | 0.092 |
| 11 | rs489574 | G | 0.777 | 45 | 622 | 0.644 | 0.804 | 5.19×10^-4^ | 0.670 | 0.819 | 0.441 | 0.218 | 0.787 | 0.406 |
| 15 | rs6493153 | A | 0.716 | 59 | 661 | 0.992 | 0.641 | 1.86×10^-14^ | -1.590 | 3.448 | -0.504 | 0.179 | 0.753 | 0.704 |
| 15 | rs4332691 | C | 0.908 | 57 | 649 | 0.526 | 0.990 | 3.41×10^-108^ | 1.005 | 0.667 | 1.104 | 0.874 | 0.929 | 0.117 |
| 17 | rs11079428 | T | 0.691 | 50 | 657 | 0.260 | 0.799 | 1.65×10^-33^ | 0.671 | 0.548 | 0.504 | 0.218 | 0.777 | 0.740 |
| 22 | rs71785313 | DEL | 0.122 | 36 | 610 | 0 | 0.150 | 7.43×10^-4^ | NA | NA | -0.380 | 0.231 | NA | 0.593 |

^a^ We tested the allele frequencies in AF_0_ (the stratum with zero copies of African ancestry) and AF_2_ (the stratum with two copies of African ancestry) using the test of proportions.

^b^ We tested for effect homogeneity by Welch’s *t*-test using the effect sizes (β) and standard errors (SE) for the stratum with zero copies of African ancestry and the stratum with two copies of African ancestry.

^c^ We tested for an admixture effect by regressing the phenotype on local ancestry, adjusting for age, global ancestry, and sex.
