# Supplementary material for: Multiple Loci Associated with Renal Function in African Americans
Source: PLoS One. 2012 Sep 13;7(9):e45112. doi: 10.1371/journal.pone.0045112 (PMC3441677; doi:10.1371/journal.pone.0045112)
Supplement: Table S3 — Significant secondary associations. (DOC) [file pone.0045112.s008.doc]

**Supplementary Table S3**. Significant secondary associations.

| **Chr** | **Gene** | **SNP** | **Position (bp)** | **Coded Allele** | **Allele Frequency** | **β_meta_** | **SE_meta_** | **Adjusted *P*** | **R^2^** |
| --- | --- | --- | --- | --- | --- | --- | --- | --- | --- |
| 1 | *PSMA5* | rs17586966 | 109757092 | T | 0.963 | 1.059 | 0.390 | 0.024 | 0.0066 |
| 3 | *TFDP2* | rs6440052* | 143141931 | T | 0.669 | 0.450 | 0.153 | 0.016 | 0.0074 |
| 3 | *TFDP2* | rs12494971* | 143147034 | C | 0.980 | -1.670 | 0.575 | 0.019 | 0.0090 |
| 15 | *WDR72* | rs732975 | 51737682 | G | 0.820 | 0.561 | 0.182 | 0.020 | 0.0076 |

* For these two SNPs, the pairwise linkage disequilibrium $r^{2}=0.0395$.
